# Phosphoproteomic Analysis of KSHV-Infected Cells Reveals Roles of ORF45-Activated RSK during Lytic Replication

**DOI:** 10.1371/journal.ppat.1004993

**Published:** 2015-07-02

**Authors:** Denis Avey, Sarah Tepper, Wenwei Li, Zachary Turpin, Fanxiu Zhu

**Affiliations:** Department of Biological Science, Florida State University, Tallahassee, Florida, United States of America; Wistar Institute, UNITED STATES

## Abstract

Kaposi’s Sarcoma-Associated Herpesvirus (KSHV) is an oncogenic virus which has adapted unique mechanisms to modulate the cellular microenvironment of its human host. The pathogenesis of KSHV is intimately linked to its manipulation of cellular signaling pathways, including the extracellular signal-regulated kinase (ERK) mitogen-activated protein kinase (MAPK) pathway. We have previously shown that KSHV ORF45 contributes to the sustained activation of both ERK and p90 ribosomal S6 kinase (RSK, a major functional mediator of ERK/MAPK signaling) during KSHV lytic replication. ORF45-activated RSK is required for optimal KSHV lytic gene expression and progeny virion production, though the underlying mechanisms downstream of this activation are still unclear. We hypothesized that the activation of RSK by ORF45 causes differential phosphorylation of cellular and viral substrates, affecting biological processes essential for efficient KSHV lytic replication. Accordingly, we observed widespread and significant differences in protein phosphorylation upon induction of lytic replication. Mass-spectrometry-based phosphoproteomic screening identified putative substrates of ORF45-activated RSK in KSHV-infected cells. Bioinformatic analyses revealed that nuclear proteins, including several transcriptional regulators, were overrepresented among these candidates. We validated the ORF45/RSK-dependent phosphorylation of several putative substrates by employing KSHV BAC mutagenesis, kinase inhibitor treatments, and/or CRISPR-mediated knockout of RSK in KSHV-infected cells. Furthermore, we assessed the consequences of knocking out these substrates on ORF45/RSK-dependent regulation of gene expression and KSHV progeny virion production. Finally, we show data to support that ORF45 regulates the translational efficiency of a subset of viral/cellular genes with complex secondary structure in their 5’ UTR. Altogether, these data shed light on the mechanisms by which KSHV ORF45 manipulates components of the host cell machinery via modulation of RSK activity. Thus, this study has important implications for the pathobiology of KSHV and other diseases in which RSK activity is dysregulated.

## Author Summary

Kaposi’s sarcoma-associated herpesvirus (KSHV) is a human tumor virus which hijacks the host signaling pathways in order to maintain persistent infection. We previously discovered that the KSHV protein ORF45 binds to and activates the cellular kinase RSK (p90 ribosomal S6 kinase), and that this activation is vital for optimal KSHV gene expression and virion production. Here, we performed a phosphoproteomic analysis of KSHV-infected cells to further characterize the specific substrates of ORF45-activated RSK. Bioinformatic analyses provided insights into the functional roles of these substrates. We verified the ORF45/RSK-dependent phosphorylation of a subset of these substrates by various means. Finally, we used genome editing to knock out RSK, as well as several cellular substrates identified by our screening, and characterized the consequent effect(s) on regulation of gene expression and virion production. Thus, this work further elucidates one of the key signaling nodes modulated by KSHV, and implicates ORF45-mediated activation of RSK in the regulation of viral and host gene expression during KSHV lytic replication.

## Introduction

The dysregulation of kinase signal transduction pathways is the basis for a variety of illnesses, including infectious diseases and multiple forms of cancer. Kaposi’s sarcoma-associated herpesvirus (KSHV), or human herpesvirus 8 (HHV-8), is a human oncogenic virus and the etiological agent of primary effusion lymphoma, multicentric Castleman’s disease, and Kaposi’s sarcoma (KS) [1–3]. The incidence and severity of these diseases is more pronounced in immunocompromised individuals, including organ transplant recipients and HIV/AIDS patients [4,5]. KS remains the most common AIDS-associated malignancy, and there is substantial experimental and epidemiological evidence to suggest a co-regulatory relationship between KSHV and HIV [6–10]. As obligate intracellular parasites, KSHV and other viruses must modulate their hosts’ cellular signaling pathways in order to evade host antiviral immune responses, express viral genes and efficiently produce and disseminate progeny virions. The remarkable ability of herpesviruses to establish lifelong infection is due to their distinctive life cycle, comprised of a latent replicative cycle with periodic reactivation of lytic replication. Several KSHV genes, both latent and lytic, have been shown to manipulate various cellular signal transduction pathways (reviewed in [11,12]).

We have previously shown that expression of the KSHV lytic protein ORF45 causes sustained activation of p90 ribosomal S6 kinase (RSK), and that this activation is critical for optimal lytic gene expression [13,14]. Importantly, a single point mutation of ORF45 (F66A) abolishes binding to and activation of RSK [15]. To assess the significance of the ORF45/RSK signaling axis to KSHV replication, we introduced the F66A point mutation into the KSHV genome and found that upon lytic reactivation, this mutant is deficient in RSK activation. This results in reduced phosphorylation of putative RSK substrates, decreased lytic gene expression, and sub-optimal progeny virion production [15].

One potential explanation for this phenomenon is that ORF45-activated RSK modulates the activities of proteins with roles in translational regulation. This idea is supported by our previous finding that ORF45/RSK induces the phosphorylation of eukaryotic translation initiation factor 4B (eIF4B), thereby increasing its assembly into the translation initiation complex, which is important for efficient lytic replication [16]. Our results also suggest a role for ORF45-activated RSK in transcriptional regulation, which is supported by the work of Karijolich et al., who recently showed that ORF45-mediated RSK2 activation transcriptionally activates the HIV-1 long terminal repeat (LTR) [17]. This is reminiscent of previous observations that ORF45 synergizes with HIV Tat to transcriptionally activate the HIV-1 LTR [7]. This is significant because HIV co-infection increases the incidence of KS 10,000-fold, an increase which cannot be explained solely by HIV-induced immunodeficiency, and is likely attributable to direct interactions between the two viruses [5,18–20]. Furthermore, work by Chang and Ganem has shown that ORF45 expression in KSHV-infected lymphatic endothelial cells contributes to RSK-dependent mTOR activation, sensitizing cells to rapamycin-induced apoptosis [21,22]. Taken together, these studies point to an integral role for ORF45-mediated RSK activation in KSHV replication and pathogenesis.

We postulate that ORF45 functions through regulatory mechanisms as diverse as the functional roles of RSK substrates, by affecting various cellular processes such as growth, proliferation, survival/apoptosis, cell cycle regulation, transcription and translation [23,24]. Here, we show widespread increases in protein phosphorylation following the induction of KSHV lytic reactivation at a putative RSK phosphorylation motif (RxRxxS*/T*) [25,26]. Furthermore, a subset of these changes was found to be dependent on ORF45-mediated RSK activation. We performed a mass-spectrometry-based phosphoproteomic analysis of KSHV-infected cells and identified several hundred putative substrates of ORF45-activated RSK. The phosphorylation of putative substrates of ORF45-activated RSK, many of which play significant roles in transcriptional/translational regulation, was further validated. Pharmacological inhibitor treatment or CRISPR-mediated knockout of RSK confirmed that RSK activity is crucial for optimal KSHV lytic replication, and CRISPR-mediated knockout of several downstream substrates shed light on their significance to ORF45/RSK-dependent transactivation, as well as to KSHV progeny virion production. Finally, we discovered a new role of the ORF45/RSK/eIF4B signaling axis in the translation of mRNAs with highly structured 5’-untranslated regions (UTRs) which has important implications for KSHV pathobiology.

## Results

### KSHV lytic replication induces widespread changes in the phosphorylation of AGC kinase substrates

**Fig 1:**
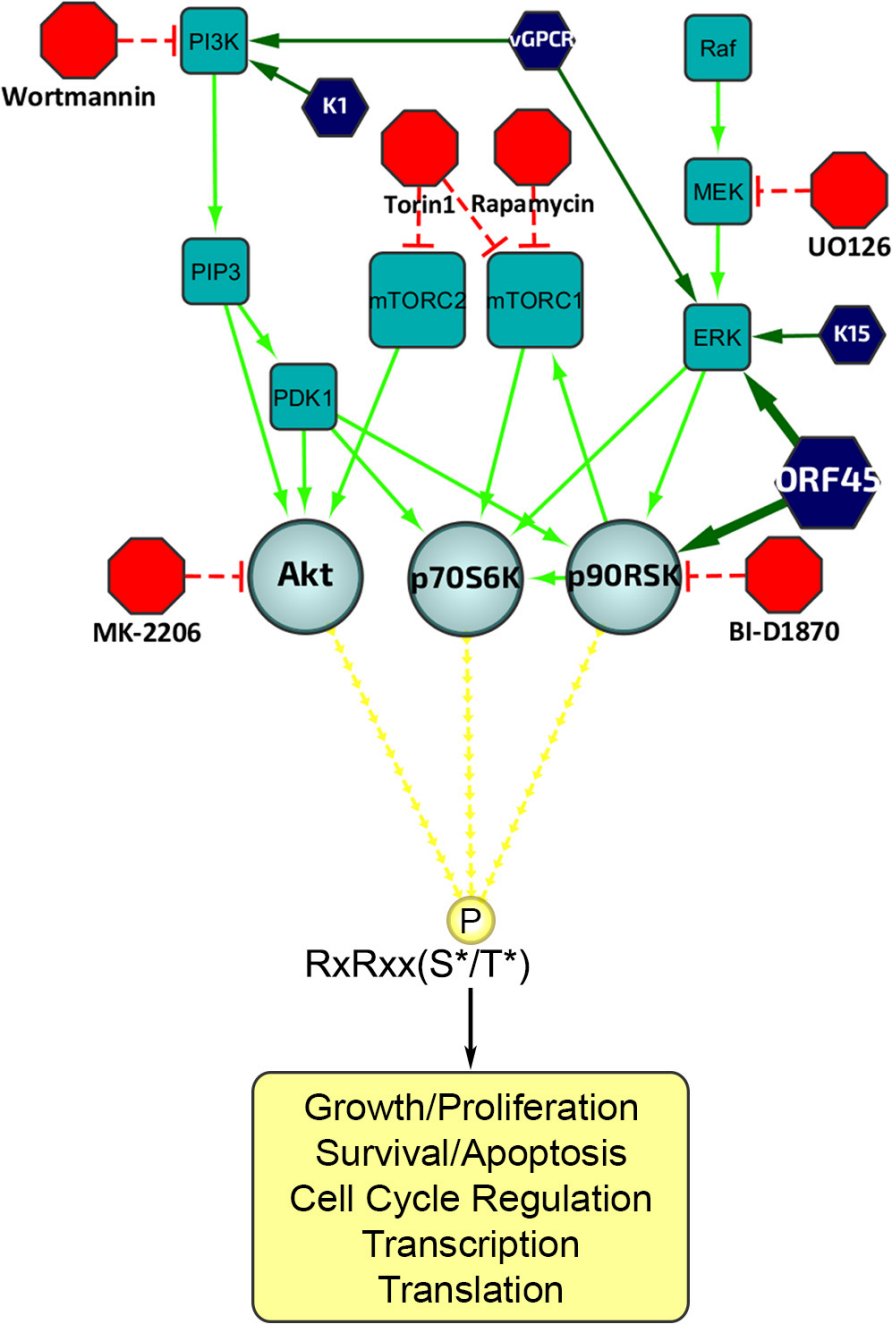
Diagram of the upstream signaling pathways that converge on activation of AGC kinases. In dark blue are the KSHV proteins that have been shown to affect various nodes of the PI3K/Akt/mTOR and/or Raf/MEK/ERK signaling pathways. In red are the kinase inhibitors used in this study. AGC kinases, including Akt, p70S6K (S6K), and p90RSK (RSK), are shown in light blue, and phosphorylate their substrates at the RxRxxS*/T* motif, leading to regulation of key biological processes.

**Fig 2:**
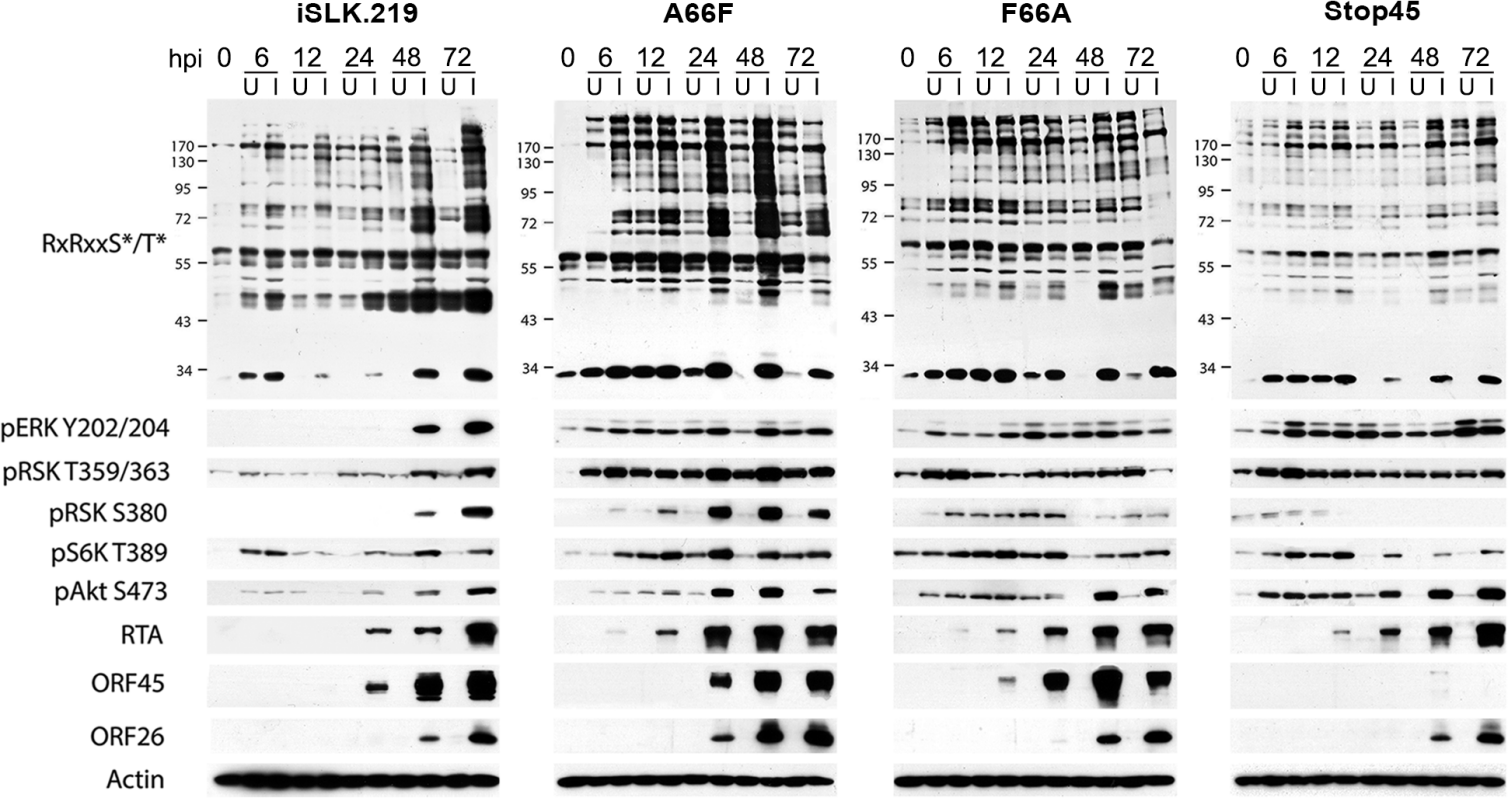
The increased phosphorylation of putative RSK substrates induced by KSHV lytic reactivation is dramatically reduced by ORF45 mutation or deletion. Stable iSLK.219 or iSLK.BAC16 cells carrying the indicated BAC (F66A, Stop45, or the F66A revertant (A66F)), were uninduced (U) or induced (I) with doxycycline and butyrate. Cell lysates were harvested at 0, 6, 12, 24, 48, and 72 hours post-induction (hpi) and analyzed by western blot using the indicated antibodies.

The ERK/MAPK and PI3K/Akt/mTOR signaling pathways are intimately involved in diverse cellular processes, and their dysregulation has been implicated in the progression of several human diseases, including KSHV-related illnesses [27–32]. A major functional mediator of ERK/MAPK signaling, RSK, as well as Akt and p70 S6 kinase (S6K), are members of the AGC kinase family known to phosphorylate their substrates at the consensus motif RxRxxS*/T* (R, arginine; S, serine; T, threonine; and x, any amino acid; Fig 1) [25,33,34]. Because of the established roles of these kinase signaling pathways in KSHV replication and pathogenesis, we set out to characterize the extent to which their substrates are phosphorylated throughout the KSHV life cycle. We first analyzed the overall pattern of phosphorylation at the RxRxxS*/T* motif upon induction of KSHV lytic reactivation in iSLK.219 cells (Fig 2; left panel). We observed dramatic differences in phosphorylation of AGC kinase substrates between induced and uninduced cells. Importantly, ERK and RSK phosphorylation at sites indicative of their activation was elevated at 48 and 72 hours post-induction (hpi), correlating well with levels of ORF45 expression. However, phosphorylation of Akt and S6K was also upregulated by KSHV lytic replication. Thus, we required an alternative system to distinguish the substrates of these other AGC kinases from those which could be phosphorylated in an ORF45/RSK-dependent manner.

### A subset of proteins is phosphorylated during KSHV lytic replication in a manner dependent on KSHV ORF45-mediated activation of RSK

Since we have previously shown that ORF45 binds to and activates RSK, and that RSK activity is important for efficient KSHV lytic gene expression, we were curious as to the significance of ORF45-mediated RSK activation to the observed differences in phosphorylation induced by KSHV. Notably, a single point mutation in ORF45 (F66A) abolishes the binding to and activation of RSK [15]. We used BAC mutagenesis to introduce this mutation into the KSHV genome in BAC16, transfected this BAC into KSHV-negative iSLK cells, and selected for stably expressing cells, designated iSLK.BAC16 F66A. We also introduced the revertant mutation back to the wild-type, iSLK.BAC16 A66F. Importantly, the F66A mutation is accompanied by a ~10-fold reduction in progeny virion production [15].

As expected, the F66A mutant virus is deficient in RSK activation, as evidenced by decreased levels of pRSK (Fig 2). We reasoned that the reduced KSHV lytic gene expression and virion production of the F66A mutant could be explained by the decreased phosphorylation and consequent functional regulation of certain RSK substrates, and therefore sought to determine the contributions of KSHV ORF45-activated RSK to the differential phosphorylation induced by KSHV lytic replication. We predicted that a subset of the KSHV-induced changes in phosphorylation would be dependent on ORF45-activated RSK, and thus be diminished in cells infected by the F66A mutant virus. Indeed this was the case, as overall phosphorylation at the RxRxxS*/T* motif was dramatically decreased in iSLK.BAC16 F66A cells compared to the revertant, especially at 24 and 48 hpi (Fig 2). As expected, we observed a dramatic decrease in ERK and RSK phosphorylation in the iSLK.BAC16 F66A cells. Moreover, the levels of phosphorylation of Akt and S6K were only moderately affected by the F66A mutation, suggesting that the considerable variation in the phosphoproteomic profiles of these cells could be attributed to the difference in RSK activity. Importantly, a similar phenomenon could be induced by infection of SLK cells with a lentivirus expressing ORF45 wild-type or F66A, suggesting that ORF45 is capable of inducing phosphorylation of cellular proteins independently of other viral factors (S1 Fig).

### A phosphoproteomic screening identified proteins exhibiting differential phosphorylation upon induction of KSHV lytic replication

**Fig 3:**
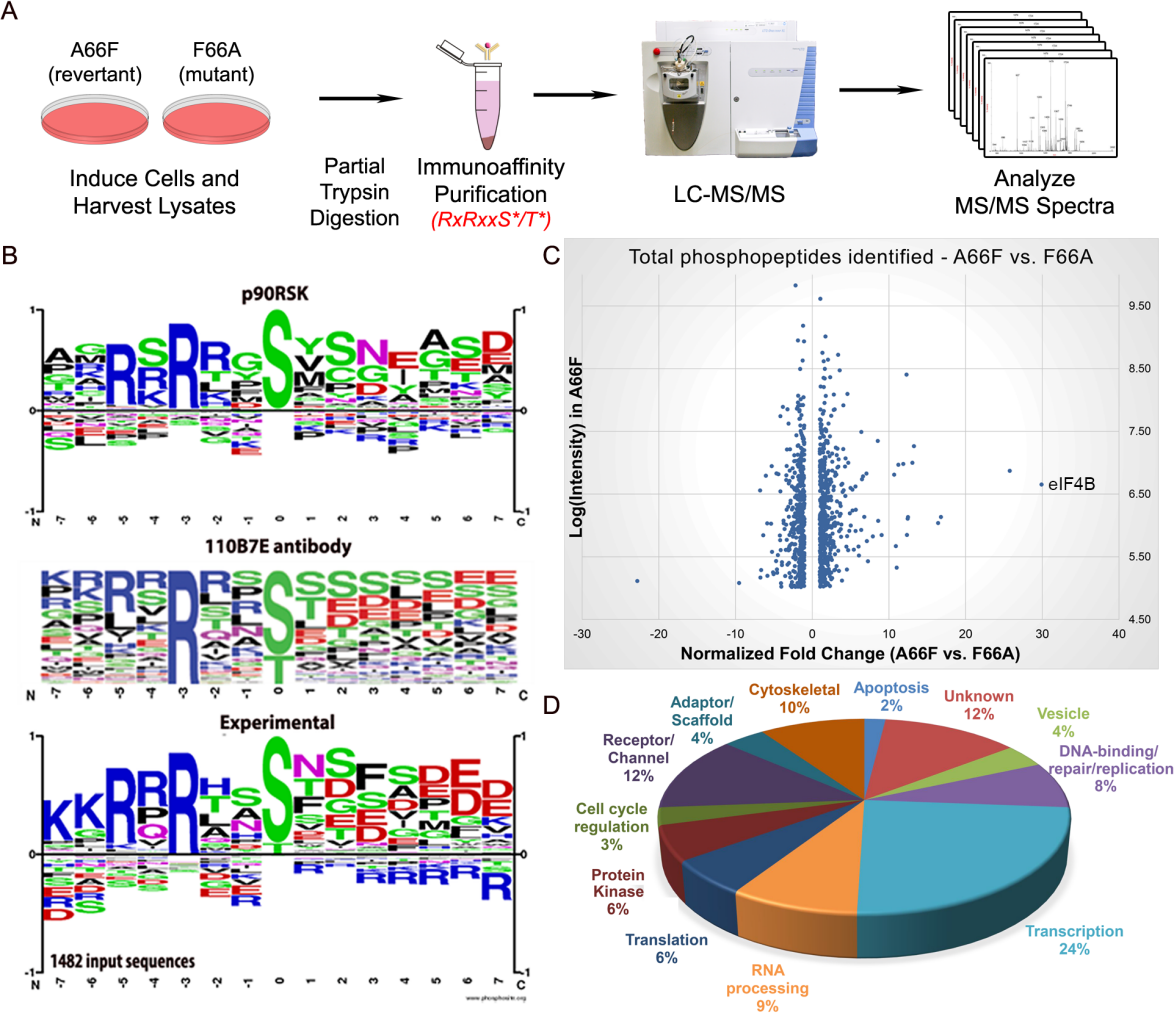
Phosphoproteomic analysis allows for the identification of putative substrates of ORF45-activated RSK during KSHV lytic replication. (A) Flowchart of the experimental design. (B) Phosphorylation motif for RSK, the 110B7E antibody used for the PhosphoScan, and the experimental data. (C) Scatterplot representation of the unique phosphopeptides identified by the screening that met the cutoff for deltaCN, intensity, and spectral counts. (D) Pie chart of the protein type/function distribution among the unique phosphopeptides with at least a 2.5-fold change (A66F:F66A).

In order to further investigate the abundant KSHV-induced phosphorylation of putative RSK substrates, we performed a phosphoproteomic screening by employing Cell Signaling Technology’s PhosphoScan service [35]. We compared the phosphoproteomic profiles of iSLK.BAC16 F66A and its revertant (A66F) in order to determine which proteins are phosphorylated in an ORF45/RSK-dependent manner. Importantly, cell lysates were immunoaffinity purified with the anti-RxRxxS*/T* motif antibody prior to analysis by LC-MS/MS (Fig 3A). A substantial amount of manual vetting was undertaken to ensure that any conclusions drawn from the MS spectra could be made with high confidence. We ignored MS spectra assigned to peptides with a DeltaCN of less than 0.1, the general rule of thumb for what constitutes a “good hit” from a SEQUEST search [36]. We identified 1482 total peptides representing 1094 unique phosphopeptides of 479 proteins that met this cutoff. Of these, 1265 phosphopeptides (85.4%) contained at least the minimal RxxS*/T* motif. The motif derived from this experimental data correlates well with the expected specificity of the antibody we used, as well as the previously characterized RSK phosphorylation motif (Fig 3B) [24–26,37].

Fig 3C shows a scatterplot representation of the unique phosphopeptides identified by the screening arranged by average intensity in the A66F (y-axis) and fold-change between the A66F and F66A (x-axis). These IDs met an additional threshold for intensity (≥100,000) and spectral counts (≥2), leaving 1000 unique peptides. Of those that had at least a 2.5-fold reduction in phosphorylation in the F66A mutant (121 unique peptides; 98 proteins), we analyzed the distribution of the major protein types represented (Fig 3D). Significantly, peptides mapping to proteins with roles in transcriptional regulation accounted for about a quarter of these putative substrates of ORF45-activated RSK. Table 1 shows a list of the 50 most significant phosphopeptides identified, arranged according to fold-change of phosphorylation. A complete list of the phosphopeptides identified can be found in the supplemental material (S1 File). We were able to identify several proteins previously described as RSK substrates, confirming the validity of the experimental design and the phosphoproteomic screening (S1 File: ‘RSK substrates’).

**Table 1:** List of genes representing the phosphopeptides identified by the phosphoproteomic screening with the highest fold-change in phosphorylation between A66F and F66A.

Gene name	Accession Number	(Log_10_) Intensity in A66F	Normalized fold-change: A66F vs. F66A	Phosphorylation Site(s)	Protein Type
EIF4B	P23588	6.65	29.91	422, 425	Translation
SETD1A	O15047	6.87	25.76	915, 916	Enzyme, misc.
LMNA	P02545	6.04	16.42	404, 406	Cytoskeletal protein
ZNF217	O75362	7.26	13.27	975	DNA binding, DNA repair/replication
SLBP	Q14493	7.00	13.07	111	RNA processing
RNF20	Q5VTR2	6.13	12.49	517	Ubiquitin conjugating system
SP100	P23497	6.11	12.46	221	Transcription
WHSC1	O96028	5.85	12.36	172	Ubiquitin conjugating system
RNF19B	Q6ZMZ0	8.40	12.30	393	Ubiquitin conjugating system
TCOF1	Q13428	6.98	11.88	107	Transcription
DDI2	Q5TDH0	6.97	11.24	194	Protease
ZNF384	Q8TF68	5.33	11.02	234	DNA binding, DNA repair/replication
ZNF217	O75362	5.67	10.91	974	DNA binding, DNA repair/replication
DNAJC2	Q99543	6.81	10.69	47	Cell cycle regulation
ARHGEF17	Q96PE2	5.85	9.06	395	G protein or regulator
GTF2F1	P35269	7.35	8.55	385	Transcription
UIMC1	Q96RL1	5.75	7.14	627	Transcription
AKT1S1	Q96B36	6.83	7.08	246	Apoptosis
POM121C	A8CG34	6.13	6.80	323, 325	Receptor/channel
RPS6	P62753	6.78	6.63	235, 236, 241	Translation
KPNA2	P52292	5.93	6.40	54	Receptor/channel
TCF3	P15923-2	5.52	6.10	529, 530	Transcription
NKRF	O15226	5.66	6.05	36	Transcription
TAF3	Q5VWG9	6.25	5.69	200	Transcription
RFC1	P35251	5.61	5.43	245	DNA binding, DNA repair/replication
DHX36	Q9H2U1	6.57	5.26	161	Enzyme, misc.
LMNA	P02545	6.00	5.04	404, 407, 414	Cytoskeletal protein
EIF4B	P23588	6.60	5.01	422	Translation
TRIT1	Q9H3H1	6.19	4.92	431	Enzyme, misc.
POM121	Q96HA1	5.91	4.85	346, 348	Receptor/channel
SRRM2	Q9UQ35	7.00	4.71	1413, 1415, 1420	RNA processing
RANBP3	Q9H6Z4	8.10	4.59	124	Adaptor/scaffold
YAP1	P46937	5.83	4.56	109, 110, 119	Transcription
GSK3B	P49841	5.96	4.53	9	Protein kinase
TAF3	Q5VWG9	7.07	4.35	199	Transcription
HNRNPL	P14866	6.47	4.33	32	RNA processing
PFKFB2	O60825	6.94	4.28	466	Phosphatase
IWS1	Q96ST2	6.56	4.10	720	Transcription
RPS6	P62753	5.12	3.89	236, 244	Translation
NDRG1	Q92597	6.01	3.80	330	Vesicle protein
BRAF	P15056	5.82	3.77	364	Protein kinase
NDRG3	Q9UGV2	5.94	3.73	331, 335	Cell development/differentiation
UBR4	Q5T4S7	8.47	3.59	1760	Receptor/channel
YAP1	P46937	6.15	3.59	127, 143	Transcription
TPX2	Q9ULW0	5.27	3.49	499	Cell cycle regulation
DDX17	Q92841	7.05	3.45	571	Transcription
H2B	P33778	6.94	3.42	37	DNA binding, DNA repair/replication
EIF4B	P23588	6.38	3.23	406	Translation
PDCD4	Q53EL6	5.66	2.93	67, 76	Apoptosis

Unique phosphopeptides identified by LC-MS/MS were filtered by deltaCN (≥0.1), intensity (≥100,000), and spectral counts (≥2), then ordered according to fold-change phosphorylation (A66F vs. F66A). The top 50 hits are shown, as well as the phosphorylation site(s) and protein type.

**Fig 4:**
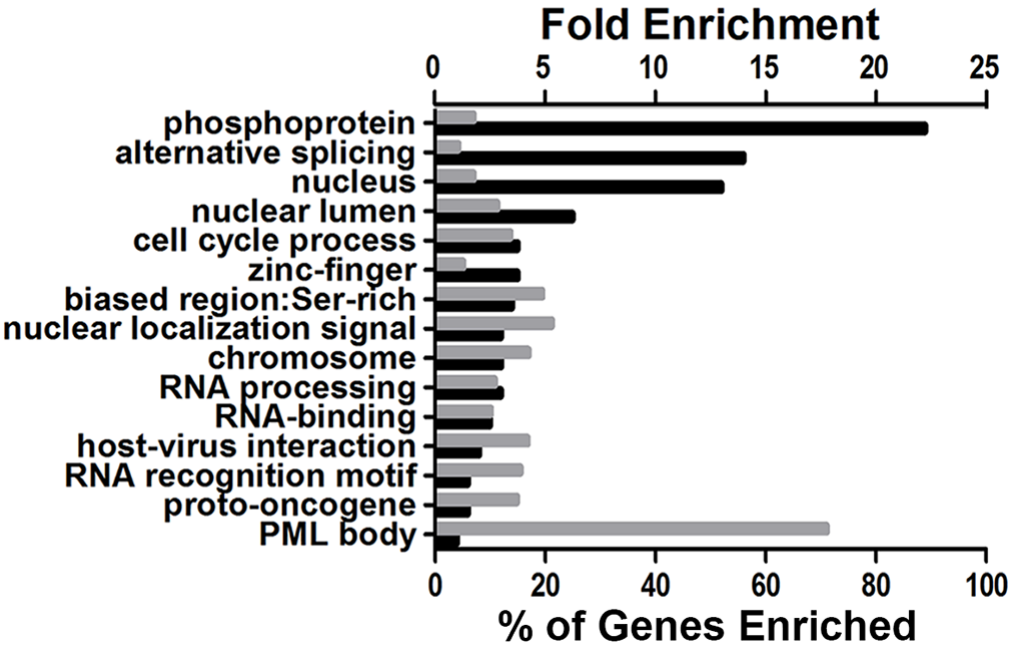
DAVID bioinformatic analysis of the phosphoproteomic screening results. The enrichment of Gene Ontology (GO) terms associated with the putative substrates of ORF45-activated RSK (121 unique phosphopeptides with ≥2.5-fold change in phosphorylation between A66F and F66A) was assessed in the background of the human proteome. The x-axes values denote the percentage of the 98 unique proteins that were enriched (black bars) or the fold enrichment (gray bars) for the indicated GO term, functional category, or protein domain. P-values were all <0.05. The exact p-values and additional information can be found in S2 File.

Data from the PhosphoScan was then subjected to DAVID bioinformatic analysis [38,39]. Our goal was to ascertain the most biologically relevant functions of potential substrates of ORF45-activated RSK. To address this, we assessed the enrichment of Gene Ontology (GO) terms, functional categories, and protein domains associated with this dataset (121 unique phosphopeptides with ≥2.5-fold change in phosphorylation) in the background of the human proteome. Fig 4 summarizes the results of this analysis, and the complete functional annotation chart can be found in S2 File. Among the most enriched categories were phosphoproteins, proteins with nuclear localization, cell cycle regulators, and proteins involved in RNA binding/processing (Fig 4). A similar analysis was performed using the total IDs from our phosphoproteomic screening as the background gene list, and yielded comparable results (S2 Fig). As a whole, these analyses are consistent with the notion that ORF45-activated RSK plays a major role in the nuclei of KSHV-infected cells, by regulating DNA/RNA-binding proteins, histones and histone-modifying enzymes, and transcription factors. Due to the low number of proteins with roles in translation whose phosphorylation was affected by F66A mutation (only two were identified in the screening–rpS6 and eIF4B), this group was not enriched in the DAVID analysis. However, because of their dramatic difference in phosphorylation, and the established effect of phosphorylation at the identified residues on these proteins’ activities, we would argue that ORF45/RSK also plays a significant role in translational regulation.

### KSHV ORF45/RSK-dependent phosphorylation of a subset of substrates was validated

**Fig 5:**
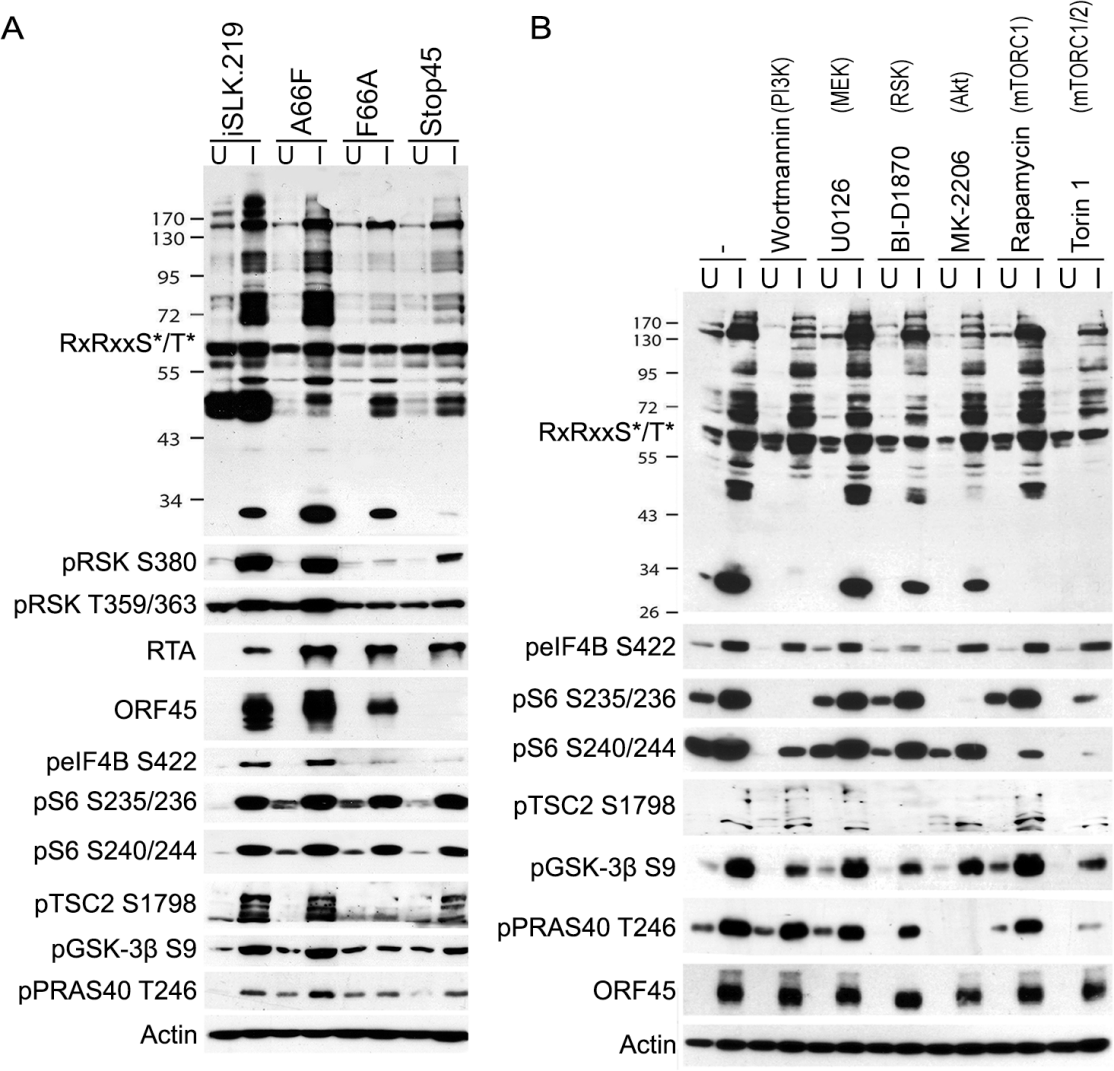
The phosphorylation of several substrates with roles in translational regulation is mediated by ORF45-activated RSK. (A) The indicated cell lines were uninduced (U) or induced (I) as described previously. Two days post-induction (dpi), cells lysates were collected and subjected to western blot analysis with the indicated antibodies. (B) Pharmacological inhibition confirms that KSHV-induced phosphorylation of eIF4B is dependent on RSK activity. iSLK.BAC16 A66F cells were uninduced (U) or induced (I) as described previously. At 46 hpi, cells were treated for 2 h with the indicated kinase inhibitor (the target of which is listed above in parentheses), then cells were harvested and lysates were analyzed by western blot with the indicated antibodies.

In order to verify the site-specific phosphorylation of the proteins identified in the PhosphoScan study, we analyzed the lysates of KSHV-infected cells by western blotting with phospho-specific antibodies. We focused on: (1) previously identified substrates of RSK, Akt, and/or S6K, (2) proteins with physiologically relevant roles to the pathogenesis of KSHV or other herpesviruses, (3) proteins with the most significant fold-changes in phosphorylation, and (4) phosphorylation sites that could be detected by commercially available phospho-specific antibodies. We were able to confirm that the site-specific phosphorylation of multiple proteins is induced by KSHV lytic replication (Fig 5A). Moreover, many of these, including eIF4B, 40S ribosomal protein S6 (rpS6 or S6), tuberous sclerosis complex (TSC2), glycogen synthase kinase 3 beta (GSK-3β), and proline-rich Akt substrate of 40 kDa (PRAS40), exhibited diminished phosphorylation in the iSLK.BAC16 F66A and/or Stop45 cells, indicating that their phosphorylation may be dependent on ORF45-activated RSK. The RSK-dependent phosphorylation of eIF4B at Ser422 is consistent with previous work published by our lab and others [16,40–42].

**Fig 6:**
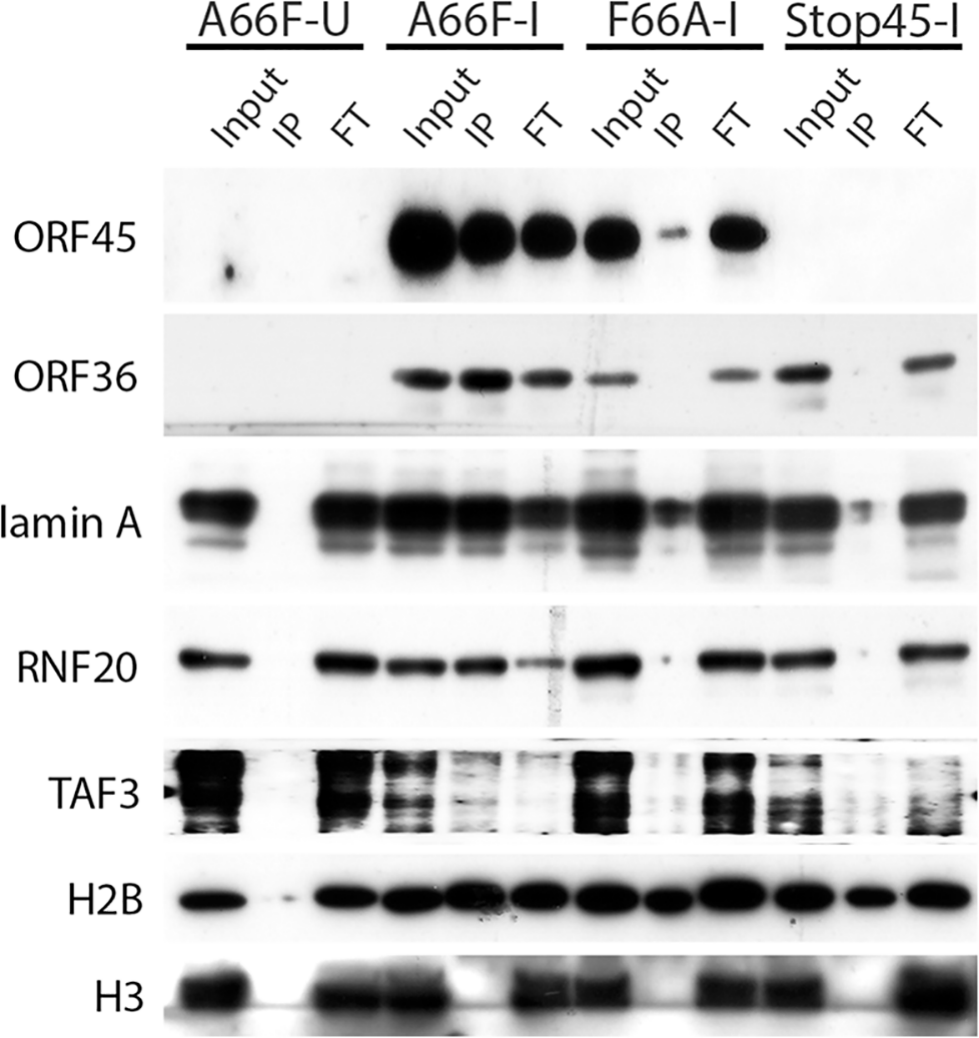
Nuclear IP further validated ORF45/RSK-dependent phosphorylation of a subset of substrates. Nuclei were isolated from the indicated cell lines at 48 hpi, then subjected to IP using anti-RxRxxS*/T* magnetic beads. The input, eluate (IP), and flow-through (FT) were analyzed by western blot with the indicated antibodies.

Because there appeared to be an enrichment of nuclear substrates of ORF45-activated RSK upon KSHV lytic reactivation (Table 1, Fig 4), and phospho-specific antibodies were unavailable, we sought to validate these putative substrates by an alternative approach. We employed nuclear fractionation of iSLK.BAC16 A66F, F66A, and Stop45 cells at 2 dpi, followed by immunoprecipitation with magnetic beads conjugated to the anti-RxRxxS*/T* motif antibody. Using this approach, we found that viral ORF45 and ORF36, and cellular Lamin A, RNF20, and TAF3 are all phosphorylated in an ORF45/RSK-dependent manner (Fig 6). While H2B phosphorylation was robustly induced by lytic reactivation, it was only mildly reduced by ORF45 mutation or deletion. Significantly, all of these substrates are known or suspected to be involved in transcriptional regulation. The phosphorylation of ORF45 itself was further confirmed by immunoprecipitation with an anti-ORF45 antibody (S3 Fig).

### Pharmacological RSK inhibition or CRISPR-mediated RSK knockout reduces RSK substrate phosphorylation and progeny virion production

To reinforce our conclusion that RSK activity is required for the differential phosphorylation of a subset of proteins during KSHV lytic replication, and to delineate the involvement of other AGC kinases, we treated KSHV-infected iSLK.BAC16 A66F cells with a panel of kinase inhibitors, including the RSK inhibitor BI-D1870 (Fig 5B) [43]. The apparent change in the pattern of overall phosphorylation at the RxRxxS*/T* motif upon treatment with inhibitors of RSK, Akt (MK-2206), or mTOR (Rapamycin and Torin 1) hints at unique and shared substrates of AGC kinases. The lack of any discernable consequence of the MEK inhibitor U0126 (compare lanes 2 and 6) is consistent with ORF45 activating the downstream ERK and RSK independently of MEK [13]. In congruence with previous results, we found that the phosphorylation of eIF4B, TSC2, GSK-3β, and PRAS40 were all reduced by RSK inhibition. The phosphorylation of eIF4B was especially and uniquely sensitive to treatment with the RSK inhibitor. In contrast, the sensitivities of S6 (to MK-2206, Rapamycin, and Torin 1) and PRAS40 (to MK-2206 and Torin 1) suggest that in this system of KSHV lytic reactivation they are predominantly phosphorylated by S6K/Akt and Akt, respectively. The mobility shift of ORF45 upon treatment with BI-D1870 is supportive of its RSK-dependent phosphorylation. The phosphorylation of TSC2 at Ser1798 also appears to be highly sensitive to RSK inhibition, which is consistent with previous findings [44]. Moreover, since this phosphorylation is known to relieve TSC2-mediated inhibition of mTOR, the implication is that this is a mechanism by which KSHV ORF45-activated RSK regulates Akt/mTOR/S6K activity [44]. We consistently observed multiple bands using the anti-pTSC2 Ser1798 antibody, which we believe represent differential extents of PTMs.

**Fig 7:**
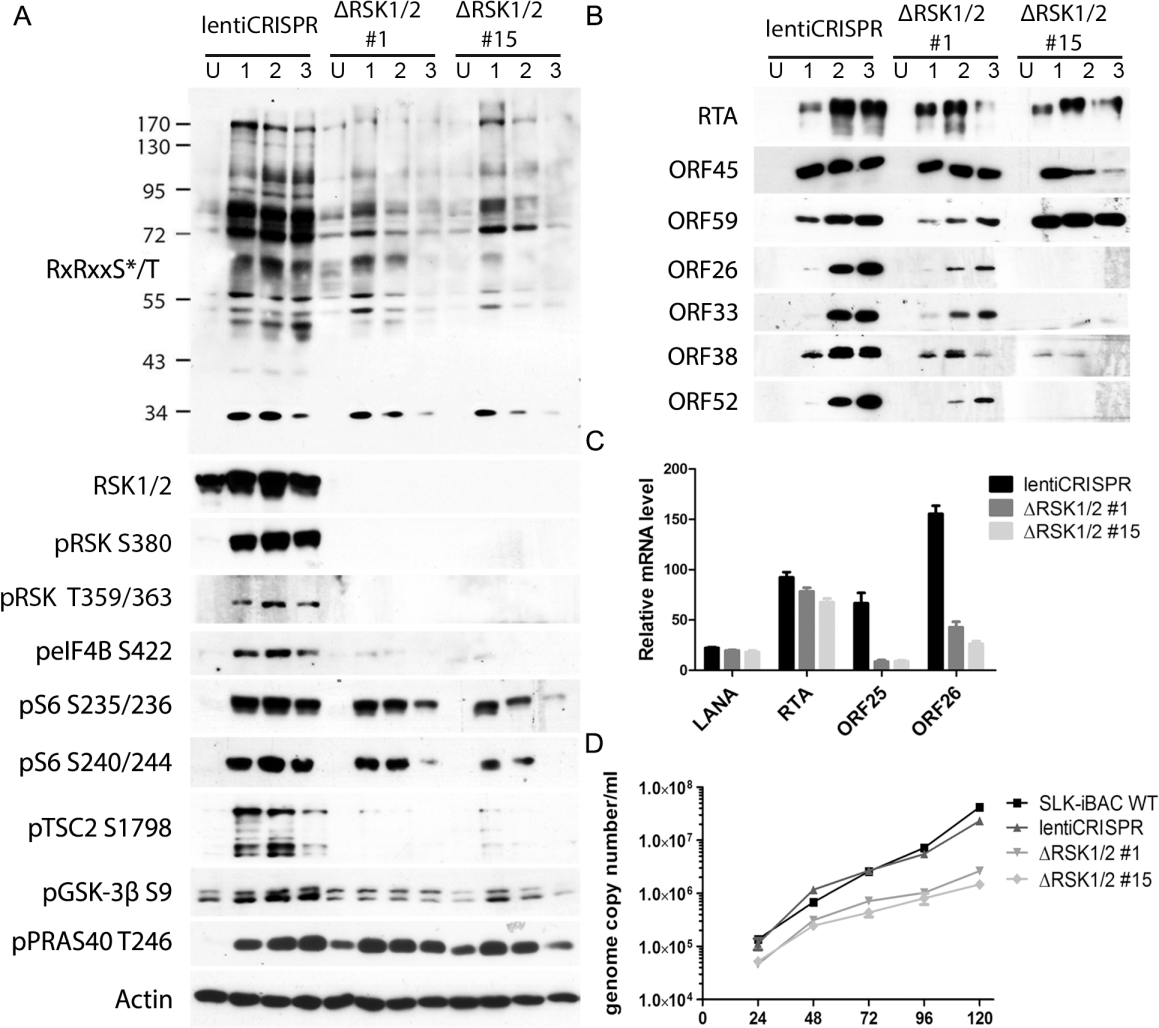
CRISPR/Cas9-mediated knockout of RSK1/2 results in the reduction of RSK substrate phosphorylation, viral late lytic gene expression, and virion production. (A and B) SLK-iBAC cells transduced with empty lentiCRISPR or single cell clones with RSK1/2 knockout (#1 and #15) were uninduced (U) or induced (I) with dox/butyrate, and cells were harvested at the indicated dpi. Lysates were analyzed by western blot with the indicated antibodies. (C) The indicated cell lines were induced as in (A and B). Twenty-four hpi, cells were harvested and RNAs were extracted, reverse transcribed, and subjected to qRT-PCR analysis using the indicated primers. Relative mRNA level is shown normalized to β-Actin. (D) KSHV progeny virion production is compromised by RSK knockout. SLK-iBAC cells were induced as described in (A and B). At the indicated hpi, media were collected from cells, extracellular virion DNA was extracted, and viral genome copy number was measured by qPCR.

We previously found that pharmacological inhibition or siRNA-mediated RSK1/2 knockdown in 293T [13] or BCBL-1 [14,16] cells suppressed TPA-induced KSHV lytic reactivation, as well as phosphorylation of eIF4B [16]. To improve and expand upon our previous studies, we knocked out both RSK1 and RSK2 in a new system, SLK-iBAC cells, using the CRISPR/Cas9 lentiviral delivery method [45]. SLK-iBAC cells are derived from the parental SLK cell line, but are stably transfected with a BAC16 mutant (iBAC) that has been engineered to express RTA from a doxycycline-inducible promoter, thereby allowing for robust lytic reactivation in a wide range of transfection-competent cell lines and negating the need for exogenous RTA induction and puromycin selection (see Materials and Methods). We induced lytic reactivation in two single cell clones with RSK1/2 knocked out, as well as in cells transduced with the empty lentiCRISPR, and compared phosphorylation at the RxRxxS*/T motif. We observed dramatically reduced phosphorylation at this motif as a consequence of RSK knockout, similar to that seen upon ORF45 mutation or pharmacological inhibition (Fig 7A). Additionally, although inducibility of these cells was not dramatically affected, late lytic protein levels were substantially reduced by RSK1/2 ablation (Fig 7B). The noticeable difference in KSHV late lytic protein levels between the two clones could be attributed to the slightly lower induction efficiency of clone #15 (Fig 7B; compare RTA level of clone #1 and #15). Relative mRNA levels of ORF25 and ORF26 (capsid components) were significantly lower in RSK knockout cells, whereas that of LANA or RTA were not dramatically affected, confirming that late lytic gene expression is compromised by RSK knockout (Fig 7C). We also measured extracellular viral genome copy number during a time course of lytic reactivation and found that RSK knockout significantly inhibits KSHV progeny virion production (Fig 7D). There is a ~10-fold decrease in virion production by 5 dpi, which is comparable to the defect of the ORF45 F66A mutant virus, and consistent with that observed upon treatment with the RSK inhibitor BI-D1870 [15].

### Assessment of the roles of ORF45/RSK substrates in the regulation of gene expression and progeny virion production

We next attempted to ascertain the contributions of the various transcriptional regulators identified by our screening in ORF45/RSK-dependent transactivation. To do so, we adopted a luciferase reporter system recently used by Karijolich et al. to describe an ORF45/RSK2-dependent mechanism for increased transactivation of the HIV-1 LTR [18]. We performed a CRISPR screen using both NH1 (containing the integrated HIV LTR fused to firefly luciferase (FL)) and NH2 (containing the LTR-FL, as well as constitutive expression of HIV Tat) cells. We targeted RSK1, RSK2, and each putative RSK substrate identified by our screening with a >10-fold difference in phosphorylation and/or a well-established role in transcriptional regulation (14 total). We then generated stably transduced knockout cell lines and performed luciferase assays in the presence and absence of exogenous ORF45 expression. Thus, the readout for this assay should give an indication of which genes are essential for ORF45-mediated transactivation of the HIV LTR, a phenomenon that is physiologically relevant in the context of KSHV/HIV co-infection in natural populations [6–10].

**Fig 8:**
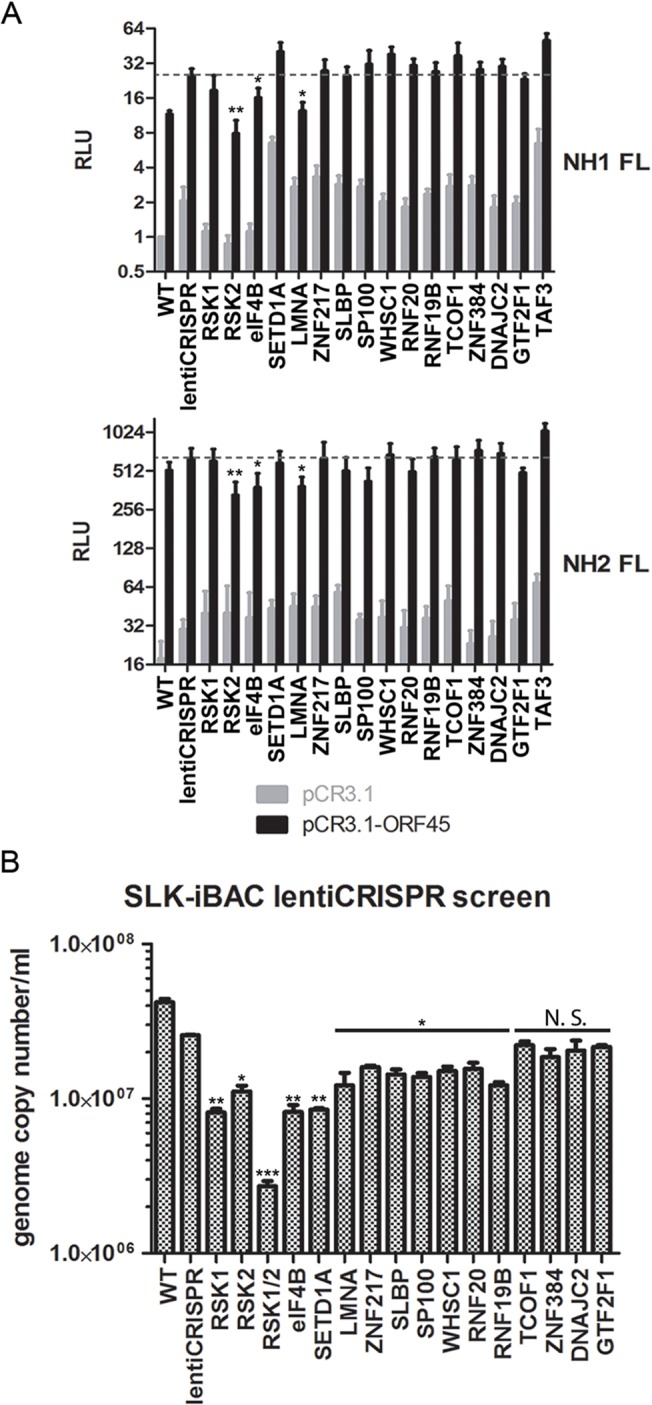
CRISPR screen characterizes the contributions of putative RSK substrates to ORF45/RSK-mediated transactivation and KSHV progeny virion production. (A) NH1 (top) and NH2 (top) reporter cells were stably transduced with the indicated lentiCRISPR, transfected with pCR3.1 or pCR3.1-ORF45, and subjected to luciferase assays. Shown are the relative light units (RLU) for firefly luciferase (FL) normalized to the NH1 FL WT sample. Values shown are the average of three biological replicates. (B) SLK-iBAC cells were stably transduced with the indicated lentiCRISPR, then induced as described previously. Extracellular virions were collected from the media at 5 dpi, and virion DNA was extracted and quantified by qPCR. Values shown are the average of three technical replicates from each of two biological replicates. Knockout of TAF3 in SLK-iBAC cells was not viable. (A and B) For RSK substrate knockout, values shown are the average of knockout cells generated from two independent gRNAs. Significance compared to empty lentiCRISPR sample: *p < 0.05; **p < 0.01; ***p <0.001; N.S.–not significant.

In concordance with the findings of Karijolich et al. [18], we found that knockout of RSK2, but not RSK1, significantly diminished the ORF45-dependent induction of luciferase expression (Fig 8A). Of the putative RSK substrates analyzed, only eIF4B or LMNA knockout had a significant effect on ORF45-mediated LTR transactivation (Fig 8A). We next performed the same CRISPR screen in SLK-iBAC cells, then induced lytic reactivation and measured progeny virion production. We detected a significant, albeit mild reduction for most genes assessed (Fig 8B). The observed effect supports the hypothesis that multiple substrates downstream of ORF45-activated RSK contribute to the optimal efficiency of the KSHV lytic cycle.

### The ORF45/RSK/eIF4B signaling axis is critical for efficient translation of mRNAs with highly structured 5’ untranslated regions (UTRs)

**Fig 9:**
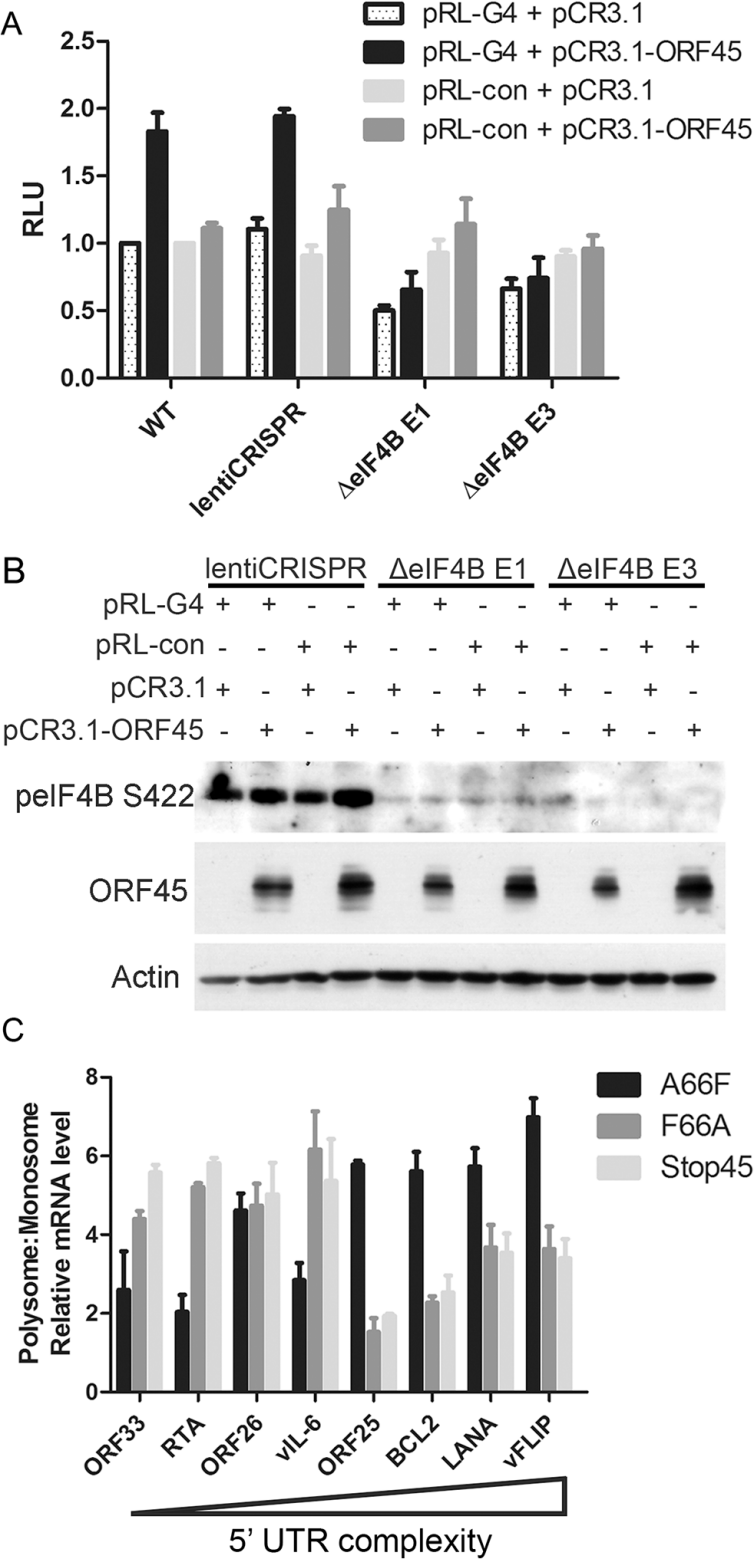
ORF45/eIF4B-dependent translational control of mRNAs with complex 5’ UTR structure. (A and B) 293T WT cells or cells stably transduced with the indicated lentiCRISPR were transfected with the indicated plasmids, as well as HCV IRES-FL (firefly luciferase), and subjected to luciferase assays (A) or western blot analysis (B) at 24 hpt. Values for (A) are represented as the renilla/firefly luciferase ratio normalized to the WT sample. (C) iSLK.BAC16 A66F, F66A, or Stop45 cells were uninduced or induced with dox/butyrate, and cells were harvested at 48 hpi. Lysates were fractionated by sucrose gradient centrifugation and RNAs were extracted. RNAs associated with either monosome or polysome fractions were pooled, reverse transcribed, and subjected to qRT-PCR using the indicated primers. Values shown are for mRNA level relative to the uninduced control, as a ratio of polysome:monosome (normalized to β-Actin). Genes are arranged in order of decreasing minimum free energy (left to right), as predicted by RNAFold software [109].

All of our data indicated that eIF4B is robustly phosphorylated during viral lytic reactivation, and that it represents one of the most specific and functionally significant substrates of ORF45-activated RSK. Thus, we aimed to further characterize the role(s) of the ORF45/RSK/eIF4B signaling axis in translational regulation. Recent reports that the RNA helicase eIF4A regulates the translation of mRNAs with G-quadruplex (G4) structures in their 5’ UTR [46], in addition to the known role of eIF4B phosphorylation in stimulating the helicase activity of eIF4A [42,47,48], led us to hypothesize that eIF4B may also contribute to this phenomenon. To test this, we transfected 293T (WT or eIF4B-knockout) cells with a reporter construct containing G4 structures in the 5’ UTR (pRL-G4) or random sequence with equal G/C content (pRL-con) in the presence or absence of ORF45 overexpression (Fig 9A and 9B). Interestingly, we found that ORF45 significantly increased luciferase activity of pRL-G4, and that loss of eIF4B almost completely abrogated this effect (Fig 9A and 9B). The ORF45-dependent induction of luciferase activity of the negative control (pRL-con) is mild by comparison and insensitive to eIF4B knockout. Thus, this difference probably represents transcriptional regulation by ORF45, whereas the more significant effect on the G4-containing reporter can be attributed primarily to translational regulation.

The apparent role of ORF45/RSK/eIF4B signaling in translational regulation led us to ask whether this pathway facilitates the translation of a specific subset of viral/cellular genes. To address this, we performed sucrose density gradient fractionation and polysome profiling of iSLK.BAC16 A66F, F66A, and Stop45 cells. This technique has been used extensively to characterize translational regulation, including that mediated by eIF4B [49,50]. Furthermore, we previously showed that exogenous expression of ORF45 promotes the association of mRNAs with polysomes [16]. This is significant because mRNAs more abundant in ‘polysome’ fractions when compared to ‘monosome’ fractions are generally considered to be translated at increased rate/efficiency. We predicted that genes with highly structured 5’ UTRs would likely rely upon the ORF45/RSK-dependent activation of eIF4B for optimal translation efficiency. Indeed, we found that mRNAs of genes with higher 5’ UTR complexity (lower predicted minimum free energy (MFE)) were detected at higher levels in polysome fractions in an ORF45/RSK-dependent manner (Fig 9C). Among these were viral ORF25, LANA and vFLIP, and cellular BCL2. Altogether, these data suggest that the ORF45/RSK/eIF4B signaling axis is important for the efficient translation of a subset of viral and cellular mRNAs containing highly structured 5’ UTRs. These findings have important implications for KSHV replication and pathogenesis (discussed below).

## Discussion

KSHV relies on manipulating the host kinase signaling pathways in order to express viral genes, evade host antiviral responses, and assemble and release progeny virions with maximum efficiency. The exact mechanisms by which specific viral proteins contribute to these various aspects of KSHV lytic replication remain unclear. The elucidation of these mechanisms is of critical importance to completely understand KSHV pathogenesis and oncogenesis, and could be useful in the development of novel therapies to treat or prevent KSHV-related illnesses. The PI3K/Akt/mTOR and ERK/MAPK signaling pathways are perhaps the two most intensively studied signaling pathways with regard to KSHV. They each have well-established roles in the progression of diverse human diseases, including various cancers, and targeted inhibition of these pathways is a common therapeutic approach with expanding applications [12,29,32,51,52]. In the context of KSHV, several viral proteins have been implicated in the dysregulation of at least one of these pathways, most notably LANA [53], vGPCR [54–62], K1 [63,64], K15 [65,66] and ORF45 [13–16].

### The multiple functions of ORF45

It is clear that KSHV ORF45 performs at least four distinct functions throughout viral lytic replication: (1) It inhibits IRF7-mediated induction of type 1 interferon by acting as a more efficient substrate of TBK1/IKKε [67–69], (2) binds to and activates ERK and RSK, resulting in the differential regulation of their various downstream substrates [13,14,16], (3) binds to the kinesin-2 motor protein KIF3A to facilitate transport of virions during egress [70], and (4) binds to and stabilizes KSHV ORF33, an event that appears to be crucial for efficient tegumentation [71]. The ORF45 F66A mutant virus, which is deficient only in the second function listed above, exhibits greater than a 10-fold reduction in progeny virion production, suggesting that the activation of ERK and RSK during lytic replication is critical for optimal lytic replication [15]. Interestingly, ORF45 possesses no inherent kinase activity, but appears to sustain ERK/RSK kinase activity by preventing their dephosphorylation [14]. The activation of RSK by ORF45 has been shown to increase the phosphorylation of the RSK substrate eIF4B and thereby enhance its assembly into translation initiation complexes, ultimately contributing to the increased translation of viral mRNAs during KSHV lytic replication [16]. Here, we further illuminate the roles of ORF45 during lytic replication.

A phosphoproteomic analysis of KSHV-infected cells identified several putative substrates of ORF45-activated RSK. We found dramatic differences in the phosphorylation of diverse substrates which can be attributed to ORF45. The picture that emerges from these data substantiates the claim that the activation of RSK by ORF45 is of critical importance during KSHV lytic replication. In the following sections, we will discuss the possible consequences of the phosphorylation of specific proteins by ORF45-activated RSK.

### ORF45/RSK-dependent phosphorylation of KSHV proteins

Several viral proteins were identified by our phosphoproteomic screening, including ORF45, ORF36, ORF52, ORF2, ORF11, and ORF59, but the differences in their phosphorylation between A66F and F66A were not especially dramatic. This could be explained by the transient nature of these phosphorylation events during the lytic life cycle and the limitation that we only analyzed the phosphoproteome at one time point, 48 hpi. The increased presence of newly synthesized, virion-contained, or otherwise unmodified viral proteins in the iSLK.BAC16 A66F lysates (compared to the replication-deficient F66A mutant) may partially explain why the fold-changes in phosphorylation were not deemed significant. A phosphoproteomic screening of iSLK.219 cells (uninduced vs. 24 hpi) did identify phosphopeptides of all of the viral proteins mentioned above, which were significantly enriched in the induced sample (S1 File). This data lends credence to the supposition that multiple viral proteins are phosphorylated at putative RSK phosphorylation motifs during KSHV lytic replication.

ORF45 itself was found to be phosphorylated at residue 303, which not only falls within the putative RSK phosphorylation motif, but is directly adjacent to ORF45’s nuclear localization signal (NLS), ^297^KRKR^300^ [72]. It is well-established that phosphorylation, especially near an NLS or NES, can affect a protein’s subcellular localization [73–75]. We have previously shown the presence of distinct mobility shifts in ORF45 protein, indicative of dramatically increased phosphorylation of the protein in the cytoplasm compared to the nucleus, and postulated that both populations of ORF45 are critical for its various functions [72]. We hypothesize that ORF45 binding to and activation of RSK increases phosphorylation of ORF45 at Ser303, thereby regulating its localization during KSHV lytic replication. This would offer an attractive explanation for the ability of ORF45 to perform several diverse roles throughout lytic replication. We have confirmed that ORF45 WT, but not the F66A mutant, is phosphorylated at the RxRxxS*/T* motif during KSHV lytic replication (Figs 6 and S3).

ORF36 was another viral protein identified by the PhosphoScan, and phosphorylation at the RxRxxS*/T* motif was reduced in iSLK.BAC16 F66A cells. ORF36 is the only serine/threonine protein kinase encoded by KSHV and is conserved throughout herpesviruses [76–78]. We confirmed that ORF36 is indeed phosphorylated at its RxRxxS*/T* motif (Fig 6). Furthermore, while investigating the phosphorylation of ORF36, we discovered a ternary interaction between ORF36, ORF45, and RSK that is dependent on ORF36 kinase activity. Based on these data, we believe that ORF45 and RSK may regulate ORF36 activity during KSHV lytic replication.

### Elucidating the mechanisms by which KSHV ORF45 regulates gene expression

Epigenetic factors are known to be involved in transcriptional regulation, and many of these, including histone-modifying enzymes, have been linked to KSHV pathobiology (reviewed in [79]). Our screening identified three histone methyltransferases: SETD1A, MLL, and WHSC1 (Table 1 and S1 File). Interestingly, the difference in phosphorylation of SETD1A between iSLK.BAC16 A66F and F66A was one of the most significant changes detected (Table 1). SETD1A is responsible for the methylation of Lys-4 of Histone 3 (H3K4). H3K4 trimethylation (H3K4me3) is associated with transcriptional activation, and has been shown to be increased at the promoters of viral genes during lytic reactivation [80–82]. Also, KSHV LANA was recently found to interact with the hSet1 complex [82]. We found that knocking out Set1 significantly reduced KSHV progeny virion production (Fig 8B). We postulate that the phosphorylation of Set1 and other histone-modifying enzymes by ORF45-activated RSK represents a mechanism by which histone PTMs are regulated throughout the KSHV life cycle. Notably, we also found that phosphorylation of histone H2B is increased during lytic reactivation in a manner partially dependent on ORF45/RSK (S1 File and Fig 6). It is possible that this phosphorylation affects the interaction between H2B and KSHV LANA, and thus the maintenance of viral episomes. The finding that RSK inhibition reduces LANA binding to H2B is consistent with this notion [53].

We previously found that mRNA levels of several KSHV genes are reduced in induced iSLK.BAC16 F66A and Stop45 cells compared to their revertants, indicating a crucial role for ORF45-activated RSK in transcription of viral genes during KSHV lytic replication [15]. A more recent study offered a potential mechanistic explanation for this effect, showing that ORF45/RSK-mediated phosphorylation and accumulation of c-Fos contributes to the increased transcription of viral genes [83]. Work by Dr. Glaunsinger’s group has elucidated an additional role for ORF45/RSK2 in transcriptional activation of the HIV-1 LTR [17]. Here, we report that the phosphorylation of numerous transcriptional regulators is significantly affected by ORF45 F66A mutation (Fig 3 and Table 1). This is consistent with a major role of ORF45/ERK/RSK in the nuclei of infected cells, which appears to be conserved amongst gammaherpesviruses [84,85]. We validated the ORF45/RSK-dependent phosphorylation of Lamin A, RNF20, and TAF3 (Fig 6). Lamin A/C has well-established roles in transcriptional regulation [86,87], and phosphorylation of the nuclear lamina is particularly relevant during herpesviral life cycles, as it has been shown to promote nuclear egress of virions [88–92]. Our CRISPR/Cas9-based screenings indicate that Lamin A (LMNA) is involved in ORF45/RSK dependent transactivation (Fig 8A), and is important for optimal progeny virion production (Fig 8B). Further studies are required to determine the consequences of site-specific phosphorylation of the nuclear lamina to KSHV replication and pathogenesis.

**Fig 10:**
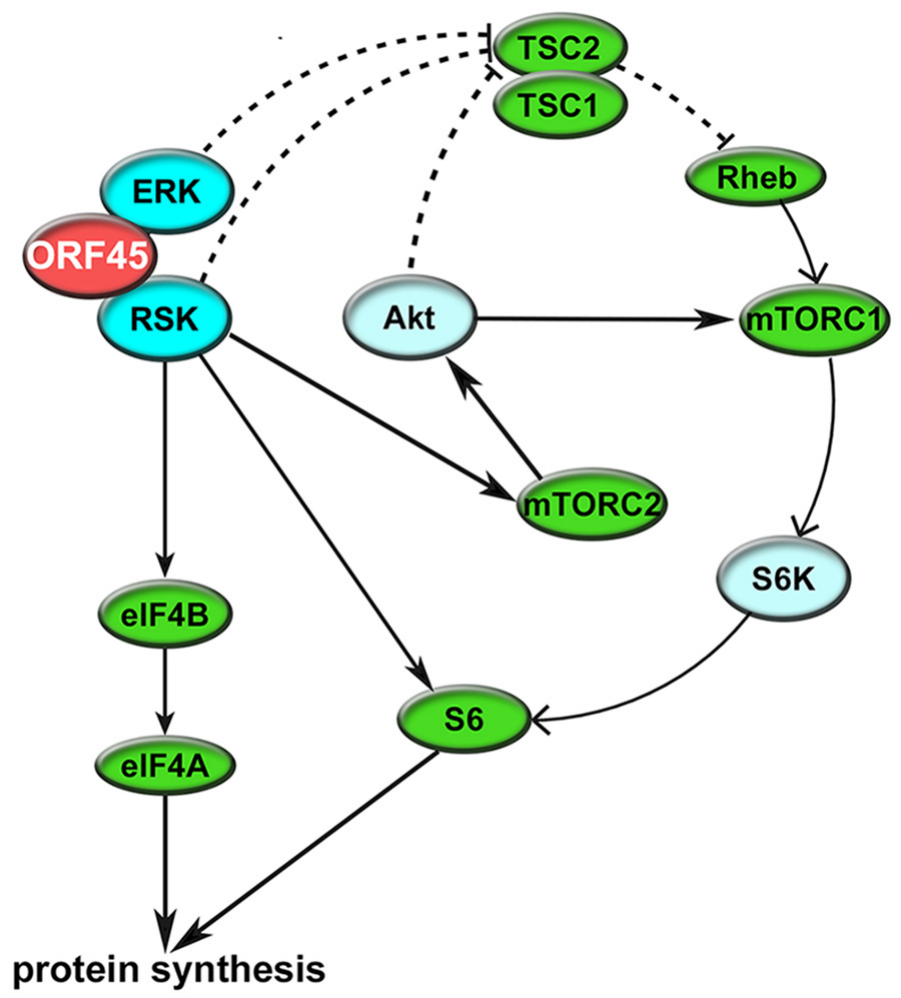
Summary of the mechanisms by which KSHV ORF45 manipulates ERK/RSK signaling to regulate translation. ORF45 regulates translation at multiple levels through direct and indirect mechanisms, including: (1) RSK-dependent phosphorylation of eIF4B and S6, (2) ERK/RSK-mediated inhibition of TSC2, relieving its inhibition of mTORC1, and (3) crosstalk with Akt/mTOR, thereby enhancing S6K-dependent phosphorylation of S6. Several other factors, such as GSK-3β, PRAS40, and PDCD4 (all of which were identified by our analyses to have decreased phosphorylation at key regulatory residues upon ORF45 F66A mutation), also contribute to this phenomenon.

We also validated the ORF45/RSK-dependent phosphorylation of multiple proteins with established roles in the regulation of translation, including eIF4B, S6, TSC2, and GSK-3β. Fig 10 shows the established nodes of PI3K/Akt/mTOR and ERK/MAPK signaling which contribute to regulation of translation. Significantly, the activity of TSC2 is of critical importance to KSHV pathogenesis [59,62]. Although several kinases can phosphorylate TSC2, and thereby contribute to mTOR activation, we show that the KSHV-induced phosphorylation of Ser1798 is dependent on ORF45-mediated RSK activation (Figs 5 and 7A). Notably, we did not detect any significant difference in the Akt-dependent phosphorylation of TSC2 at Ser939 or Thr1462 [93]. Our results also clearly implicate ORF45-activated RSK in phosphorylation of the translation initiation factor eIF4B during KSHV lytic reactivation. This finding corroborates previous work by our laboratory, in which we reported that KSHV-induced phosphorylation of eIF4B at Ser422 is insensitive to U0126 and rapamycin, and promotes progeny virion production [16]. Taken together, these results are remarkably consistent with what has recently been observed by Chang and Ganem. They showed that in KSHV-infected lymphatic endothelial cells (LECs), but not blood endothelial cells (BECs), there was an increase in pERK, pRSK, peIF4B (Ser422), pS6, and pTSC2 (Ser1798 but not Thr1462). Presumably, this is due to ORF45 expression in LECs, since siRNA-mediated ablation of ORF45 expression abolished many of these changes [21].

It has been known that phosphorylation of eIF4B at the residues identified by our MS analysis (Ser406 and Ser422) are involved in its ability to enhance the RNA helicase activity of eIF4A [42,47,48]. The Ser422 residue can be phosphorylated by Akt [42], S6K [40], and/or RSK [41,94]. However, our data show that in the context of KSHV lytic reactivation, the upregulation of eIF4B phosphorylation at this site is uniquely sensitive to RSK inhibition/ablation (Figs 5B and 7A). Interestingly, eIF4B activity has been shown to be especially critical for the translation of mRNAs with highly ordered 5’ UTRs [50,95]. Knockdown of eIF4B led to the discovery of several eIF4B-dependent transcripts that have roles in cell proliferation and survival, including c-MYC and BCL2 [50,96]. Recently, it was found that RNA G-quadruplexes are a key feature present within the 5’ UTRs of eIF4A-dependent transcripts, many of which are bona fide oncogenes [46]. This was discovered by sequencing the ribosome-protected mRNAs (ribosome profiling; Ribo-Seq [97]) of cells treated with a selective inhibitor of eIF4A. We used a luciferase reporter assay to show that ORF45 increases the translation of a G4-containing construct in a manner dependent on eIF4B (Fig 9A and 9B). It has been reported that G4 binding by EBV EBNA1 is important for viral DNA replication and episome maintenance [98]. More recently, it was found that G-quadruplexes regulate EBNA1 mRNA translation [99]. A simple sequence motif analysis identifies several putative quadruplex forming G-rich sequences (QGRS) in the KSHV genome [100]. Thus, it is likely that G-quadruplex structures are important for KSHV replication and pathogenesis. Growing evidence for the *in vivo* relevance of G4s opens up exciting prospects for the development and application of innovative diagnostics and therapeutic agents [101,102].

Altogether, studies of eIF4B and related translation initiation factors have shed light on the complexity of mammalian translational regulation, and indicated that these components represent potential targets for novel anti-cancer therapies [49]. Because many viral and cellular genes implicated in KSHV pathogenesis contain long and/or structured 5’ UTRs (for example, ORF25, ORF71 (vFLIP), ORF73 (LANA), VEGF-A, c-MYC, and BCL2), we hypothesized that ORF45/RSK-mediated phosphorylation of eIF4B is a mechanism by which KSHV can regulate the translation of a subset of mRNAs. Indeed, we see a clear correlation between the degree of complexity of 5’ UTR structure and the efficiency of ORF45/RSK-dependent translation for the viral/cellular genes we tested (Fig 9C). Ribosome profiling of eIF4B-knockout cells would allow for more in-depth and unbiased characterization of the identities and hallmarks of eIF4B-dependent transcripts, and performing this analysis in KSHV-infected cells would make it possible to determine the extent to which the ORF45/RSK/eIF4B signaling axis regulates mRNA translation during KSHV replication (Fig 10). Recent studies have contributed to our current understanding of the regulatory mechanisms governing changes in viral/host gene expression in KSHV-infected cells [79]. The development of innovative tools and techniques, coupled with unprecedented advances in sequencing technology, have facilitated the characterization of epigenetic, transcriptional, post-transcriptional, and translational levels of regulation. Comprehensive gene expression profiling of KSHV-infected cells is fundamental to further our understanding of how KSHV modulates the host cellular machinery, and, more importantly, how these changes contribute to KSHV pathogenesis. By aspiring to more completely characterize the complex workings of cellular signaling pathways, we will uncover shared and unique mechanisms of dysregulation by human pathogens, which could lead to the future development of novel, targeted therapies to prevent or treat a wide variety of human diseases.

## Materials and Methods

### Antibodies, chemicals, and vectors

Anti-RxRxxS*/T* antibody was ordered from Cell Signaling Technology (#110B7E). Anti-RTA (ORF50) monoclonal mouse antibody was given by Dr. Ke Lan (Institut Pasteur of Shanghai). Anti-ORF59 antibody was given by Dr. Robert Ricciardi. Monoclonal antibodies against ORF26, ORF52, ORF33, ORF38 and ORF45 were generated by the Florida State University hybridoma facility. pTSC2 (Ser1798) was ordered from LifeSpan Biosciences. pAkt, pS6K, pGSK-3β, and pPRAS40 antibodies, anti-RxxS/T-conjugated magnetic beads, Wortmannin (#9951), and Rapamycin (#9904) were ordered from Cell Signaling Technology. MK-2206 dihydrochloride was ordered from Santa Cruz Biotechnology (sc-364537), Torin 1 was ordered from Tocris Bioscience (#4247). The pCDH lentiviral expression construct and pPACK packaging plasmids were ordered from System Biosciences. The lentiCRISPR-puro vector was ordered from Addgene (#49535) [45]. The pRL-G4 and pRL-con vectors were graciously provided by Dr. Hans-Guido Wendel and previously described [46]. All other antibodies and chemicals used in this study have been described previously [13,14,72,103].

### Cell culture

SLK, 293T, NH1, and NH2 cells were cultured in Dulbecco's modified Eagle's medium (DMEM) containing 10% FBS and antibiotics. NH1 and NH2 cells were graciously provided by Dr. Britt Glaunsinger [17]. iSLK.219 and iSLK.BAC16 cells were cultured in DMEM containing 10% FBS, antibiotics, 450 μg/ml G418, 400 μg/ml hygromycin B and 1 μg/ml puromycin [104]. The induction conditions were as follows: Cells were seeded so that they reached 90–95% confluence one day later. The media was replaced with DMEM containing 1% FBS with or without doxycycline (0.2 μg/ml for iSLK.219; 2 μg/ml for iSLK.BAC16) and sodium butyrate (1 mM). Cells were harvested at the indicated times post-induction.

### iBAC construction

SLK-iBAC are a cell line recently developed by our laboratory derived from the parental SLK cells and stably transfected with a BAC16 mutant which contains a doxycycline-inducible RTA. This BAC contains the TET-on transactivator (Tet3G) and the tet-responsive elements (P_Tre3G_; inserted upstream of RTA). This therefore bypasses the requirement for inducible RTA expression from an exogenous source (as is the case for iSLK cells), allows for selection/maintenance of BAC-positive cells with a single antibiotic (hygromycin B; not G418 or puromycin), and should be useful for the study of KSHV latent/lytic replication in a broad range of transfection-competent cells (Li et al., manuscript in preparation). SLK-iBAC induction conditions were identical to those for iSLK.BAC16 except that the concentration of doxycycline used was 0.05 μg/ml (because the dox-inducible promoter is on the BAC, induction is much more efficient).

### Immunoprecipitation and western blot analysis

Western blot analysis was performed as previously described [13,14,16]. For immunoprecipitation of ORF45 from iSLK.BAC16 cells, we used a monoclonal anti-ORF45 antibody (8B8) conjugated to CNBr-Activated Sepharose 4B (GE Life Sciences). Clarified lysates were bound to the beads for 2 h at 4°C, washed three times each with lysis buffer and TBS, and bound complexes were eluted by boiling. For nuclear IP, cells were harvested in Tween 20 lysis buffer (25 mM Tris/Hepes pH 8.0, 20 mM NaCl, 2 mM EDTA, 1 mM PMSF, 0.5% Tween 20) for 15 min on ice, then nuclei were pelleted by centrifugation at 6,000 *× g* for 1 min at 4°C. The pellet was resuspended in Tween 20 lysis buffer containing 500 mM NaCl and incubated on ice for 15 min, then fresh lysis buffer (without NaCl) was added to bring the final salt concentration to 250 mM. This was centrifuged at 10,000 *× g* for 15 min and the supernatant was saved as the soluble nuclear extract for IP. The IP was performed according to the manufacturer’s instructions (Cell Signaling Technology). For western blot, about 20 μg of proteins were resolved by SDS-PAGE and transferred to nitrocellulose membranes. The membranes were blocked in 5% dried milk in 1× PBS plus 0.2% Tween 20 and then incubated with diluted primary antibodies for 2 h at room temperature or overnight at 4°C. Anti-rabbit or anti-mouse IgG antibodies conjugated to horseradish peroxidase (Pierce) were used as the secondary antibodies. SuperSignal chemiluminescence reagents (Pierce) were used for detection.

### ORF45 lentiviral transduction

293T cells were co-transfected with pCDH-CMV-MCS-EF1-copGFP (CD511B-1; containing either no insert, ORF45 WT, or ORF45 F66A) and the pPACK packaging plasmid mix according to the manufacturer’s instructions. Media containing pseudoviral particles were collected at 48 and 72 hpt, and clarified by centrifugation. SLK cells were transduced using a standard spinfection protocol. Briefly, lentivirus-containing media was added to SLK cells in the presence of 4 μg/ml polybrene. Cells were immediately centrifuged at 800 *× g* for 1 h at 37°C, and cultured under normal conditions for 24 h. The media was aspirated, fresh DMEM containing 1% FBS was added, and cells were cultured overnight. At 48 hpi, cells were harvested and lysates were subjected to western blot analysis.

### Phosphoproteomic analysis

Approximately 2 × 10^8^ iSLK.BAC16 F66A or A66F cells were induced as described previously. At 48 hpi (when we observed the most dramatic effect of ORF45/RSK-mediated substrate phosphorylation), cells were washed twice with PBS, and lysed in freshly prepared urea lysis buffer (20 mM HEPES pH 8.0, 9 M urea, 1 mM sodium orthovanadate, 2.5 mM sodium pyrophosphate, 1 mM β-glycerophosphate; 1.5-ml per plate). Lysates were sonicated and clarified by centrifugation, and the lysate supernatant was flash-frozen in liquid nitrogen and stored at -80°C. Samples were then sent to Cell Signaling Technology (CST) and subjected to their PTMScan discovery service [35].

The complete protocol, from sample prep to MS analysis, can be found on the CST website. Briefly, proteins in lysates were carboxamidomethylated to inactivate enzymatic activity (addition of DTT, then iodoacetamide), then digested with LysC. The digested cell lysate was acidified by addition of TFA to a final volume of 1%, and peptides were purified using a Sep-Pak C_18_ column. Subsequently, immunoaffinity purification (IAP) was performed using the PTMScan Phospho Akt Substrate Motif (RxxS*/T*) mAb Kit #5561. Peptides were concentrated and purified on ZipTip, followed by a final trypsin digestion step before liquid chromatography-tandem mass spectrometry (LC-MS/MS). Two technical replicates were run for each biological sample. The spreadsheet with the total IDs is available in S1 File.

### Bioinformatic analyses

The experimental motif (Fig 3B) was generated using the Sequence Logo Generator in the Motif & Logo Analysis Tools on PhosphoSitePlus [105]. The scatterplot in Fig 3C shows all of the unique phosphopeptides identified that met a cutoff for deltaCN (≥0.1), intensity (≥100,000), and spectral counts (≥2). The pie chart in Fig 3D clusters the 121 unique phosphopeptides that met a cutoff of 2.5-fold-change in phosphorylation (A66F:F66A), in addition to all of the aforementioned criteria, by their protein type. DAVID functional annotation was performed by uploading a list of all unique gene names with at least one phosphopeptide exhibiting 2.5-fold change in phosphorylation (A66F:F66A), and analyzing this list in the background of either the entire human genome (Fig 4) or a list of the total unique gene names identified by the PhosphoScan (S2 Fig) [38,39].

### Kinase inhibitor assays

iSLK.BAC16 cells were induced as described previously. At 46 hpi, one of the following inhibitors was added at the indicated concentration: Wortmannin (PI3K; 200 nM), U0126 (MEK; 10 μM), BI-D1870 (RSK; 10 μM), MK-2206 (Akt; 1 μM), Rapamycin (mTORC1; 10 nM), Torin 1 (mTORC1/2; 250 nM), or DMSO control. Lysates were collected 2 h later (48 hpi) and analyzed by western blotting.

### Genome editing

Potential guide RNAs (gRNAs) targeting the first exon of RSK1 (RPS6KA1; NM_002953; chr1:26856249–26901520), RSK2 (RPS6KA3; NM_004586; chrX: 20168029–20284750), and each of the 14 putative RSK substrates were analyzed using the CRISPR Design tool (crispr.mit.edu) [106]. Primers used for cloning of the target sequences chosen are included in the supplemental material (S1 Table). Double-stranded oligos were generated and cloned into the lentiCRISPRv1 vector, which was then transfected into 293T cells [45,107]. At 2, 3, and 4 d following transfection, media from cells was collected, clarified by centrifugation, and filtered through a 0.45-μM filter to collect lentiviral particles. These were used to transduce 293T, NH1, NH2, or SLK-iBAC cells in a 24-well plate at an MOI of 0.5. Cells were supplemented with media containing this lentiviral mix and 4 μg/ml polybrene, immediately centrifuged at 800 *× g* for 1 h at 37°C, and cultured under normal conditions for 24 h. The day after transduction, cells were trypsinized and grown in the presence of 1 μg/ml puromycin. Genomic DNA surrounding the Cas9 cleavage sites were extracted using QuickExtract solution, and PCR amplified using the Herculase II Fusion DNA polymerase (Agilent). Cleavage was confirmed using the SURVEYOR mutation detection kit (Transgenomic), as well as Sanger sequencing. For knockout of putative RSK substrates, including eIF4B, the pooled puromycin-resistant populations were used for further experiments. For isolation of SLK-iBAC ΔRSK1/2 clones, cells were diluted to a concentration of ~0.5 cells/100 μl, seeded to 96-well plates, and grown for 2–3 weeks in the presence of 1 μg/ml puromycin. Thirty single clones were isolated and assayed by western blot analysis for RSK1/RSK2 protein expression. Of these, two that showed complete loss of signal (#1 and #15) were chosen for further analyses.

### Sucrose density gradient fractionation and polysome analysis

The cells were washed once in cold phosphate-buffered saline (PBS) containing 100 μg/ml cycloheximide and scraped off the plate into 1 ml of the same solution. The cells were centrifuged for 10 min at 1,200 rpm and resuspended in 425 μl hypotonic lysis buffer (5 mM Tris-HCl [pH 7.5], 2.5 mM MgCl_2_, 1.5 mM KCl). The lysates were transferred to a prechilled tube, incubated with 100 μg/ml cycloheximide, 2 mM dithiothreitol (DTT), and 2 μl RNasin (40 U/μl; Stratagene), kept on ice for 5 min, and vortexed. To each 425 μl of lysate, 25 μl of 10% Triton X-100 and 25 μl of 10% sodium deoxycholate were added. The samples were vortexed again and incubated on ice for 5 min. Cell extracts were centrifuged for 5 min at 14,000 rpm; the supernatant was collected, and protein concentration was determined. Equal amounts of protein were loaded onto prechilled 10 to 50% sucrose gradients. Each gradient was formed in a Beckman centrifuge tube (14 by 89 mm) (catalog no. 3311372; Beckman Instruments, CA) using a BioComp gradient master according to the manufacturer’s instructions. The tubes were centrifuged in a Beckman SW40Ti rotor at 35,000 rpm for 3 h at 4°C. Fractions (0.5 ml) were collected and RNA concentration and quality was assessed by Nanodrop and denatured agarose gel electrophoresis. RNAs in the monosomal or polysomal fractions were extracted and analyzed by qRT-PCR (see below).

### Reverse transcription and RT-PCR

Sucrose gradient fractions were subjected to RNA extraction using TRIzol (Invitrogen). Reverse transcription was performed using a SuperScript III reverse transcriptase kit (Invitrogen) according to the manufacturer’s instructions. PCRs were carried out as described below. PCR was performed using 1 μl of RT reaction mixture in a total reaction mixture volume of 20 μl containing reverse and forward primers (1 μl of 10 μM solutions). Primer sequences used for BCL2 are TTTCTCATGGCTGTCCTTCAGGGT and AGGTCTGGCTTCATACCACAGGTT. Primer sequences for all other genes analyzed in this study were previously described [15,108].

### Quantification of extracellular virion genomic DNA by real-time qPCR

Viral DNA was isolated from supernatant medium of induced iSLK.BAC16 and derivative cells as previously described [72,103]. The DNAs were used in SYBR green real-time PCRs with KSHV-specific primers: ORF73-LCN (5’-CGCGAATACCGCTATGTACTCA-3’) and ORF73-LCC (5’-GGAACGCGCCTCATACGA-3’) with Bio-Rad C1000TM Thermal Cycler and CFX96TM Real-Time Detection System. Viral DNA copy numbers were calculated with external standards of known concentrations of serially diluted BAC16 DNA ranging from 1 to 10^7^ genome copies per reaction.

### Luciferase assay

For luciferase reporter assays in NH1/NH2 cells, cells were transduced with various lentiCRISPR constructs as described above (two unique gRNA targets per gene of interest), and maintained under puromycin selection for one week. Cells were then seeded to 24-well plates. The next day, cells were transfected using Lipofectamine 2000 according to the manufacturer’s protocol, with pCR3.1 or pCR3.1-ORF45 (200 ng), as well as pRL-TK (a renilla luciferase reporter construct; 10 ng). Cells were harvested at 24 hpt and luminescence was detected using the Promega Dual-Luciferase Reporter Assay System according to the manufacturer’s instructions. Values shown are the averages of three biological replicates. For G4 luciferase assays, 293T cells were untreated or transduced with empty lentiCRISPR or lentiCRISPR containing gRNAs to target eIF4B exon 1 (E1) or exon 3 (E3) as described above. Stable cell lines were seeded to 24-well plates and co-transfected with pCR3.1/pCR3.1-ORF45 and pRL-G4/pRL-con, as well as HCV IRES-FL (firefly luciferase; negative control). Cells were harvested and luminescence was detected as described above. Values shown are the relative ratio of Renilla luciferase (RL) to firefly luciferase (FL), averaged from three biological replicates.

## Supporting Information

S1 File CST PhosphoScan data. The raw spreadsheet obtained from the CST PhosphoScan, including all of the information acquired from the phosphoproteomic screening.
(XLSX)

S2 File Details of DAVID Bioinformatic analysis. DAVID functional annotation analyses featuring the complete list as well as clustering of GO terms, functional categories, and protein domains that were enriched among the phosphopeptides with at least a 2.5-fold change (A66F:F66A).
(XLSX)

S1 Fig ORF45 WT but not the F66A mutant induces phosphorylation of RSK substrates independently of the viral background. SLK cells were transduced with lentiviral particles containing no expression vector, ORF45 wild-type, or the F66A mutant. At 48 hpi, cells were lysed, and the lysates were analyzed by western blot with the indicated antibodies.
(TIF)

S2 Fig DAVID bioinformatic analysis. DAVID functional annotation analyses were performed using the same parameters as in Fig 4, except that the total unique IDs from the PhosphoScan were used as the background gene list.
(TIF)

S3 Fig ORF45 WT but not the F66A mutant is phosphorylated at the RxRxxS*/T* motif during KSHV lytic replication. ORF45 was immunoprecipitated from iSLK.BAC16 A66F or iSLK.BAC16 F66A cells using 8B8 monoclonal antibody as described previously [15]. The inputs, eluates, and flow-throughs were analyzed by western blot with the indicated antibodies.
(TIF)

S4 Fig NH1 and NH2 lentiCRISPR screen–Renilla luciferase. The same samples as in Fig 8A were analyzed for renilla luciferase activity.
(TIF)

S1 Table List of primers used to clone gRNAs for CRISPR screening. (XLS)

S1 References (DOCX)
