# Supplementary figures and images for: Phosphoproteomic Analysis of KSHV-Infected Cells Reveals Roles of ORF45-Activated RSK during Lytic Replication
Source: PLoS Pathog. 2015 Jul 2;11(7):e1004993. doi: 10.1371/journal.ppat.1004993 (PMC4489790; doi:10.1371/journal.ppat.1004993)

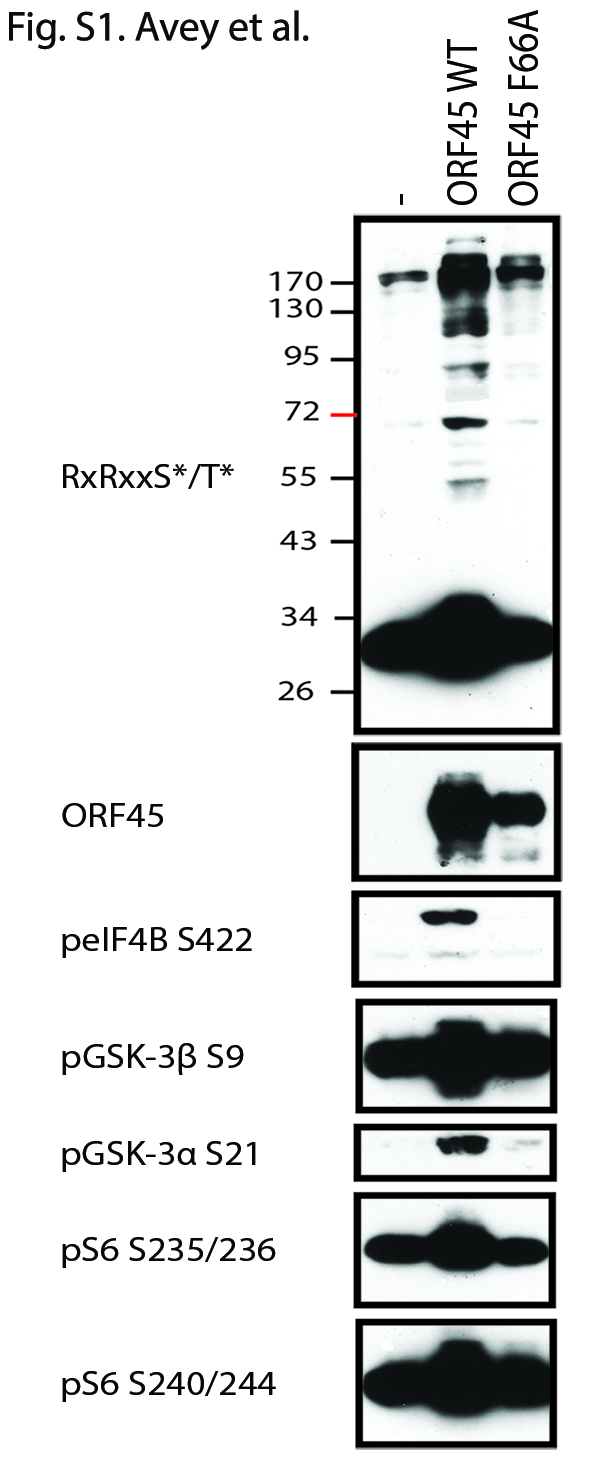

Supplement: S1 Fig — SLK cells were transduced with lentiviral particles containing no expression vector, ORF45 wild-type, or the F66A mutant. At 48 hpi, cells were lysed, and the lysates were analyzed by western blot with the indicated antibodies. (TIF) [file ppat.1004993.s003.tif]

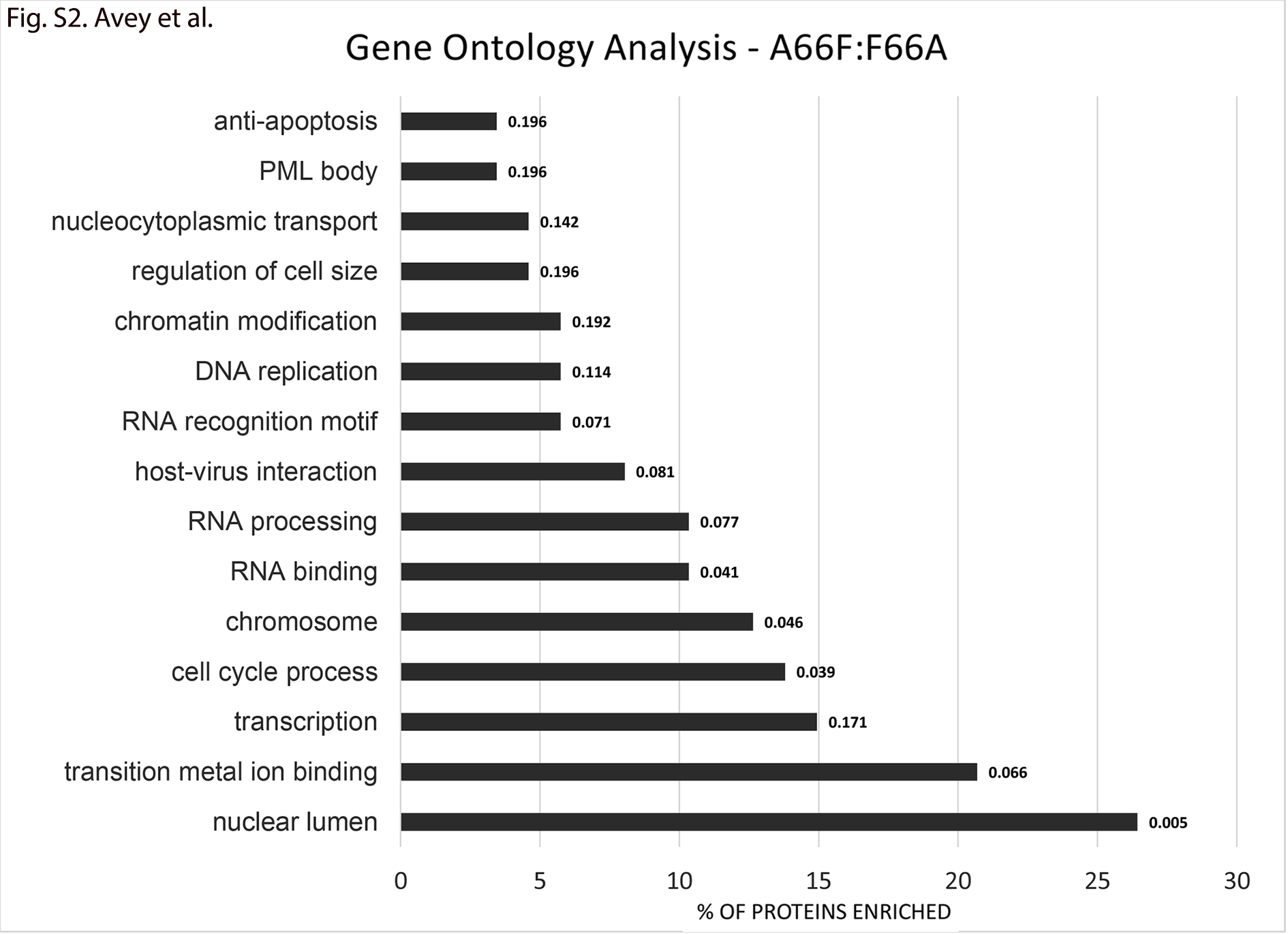

Supplement: S2 Fig — DAVID functional annotation analyses were performed using the same parameters as in Fig 4, except that the total unique IDs from the PhosphoScan were used as the background gene list. (TIF) [file ppat.1004993.s004.tif]

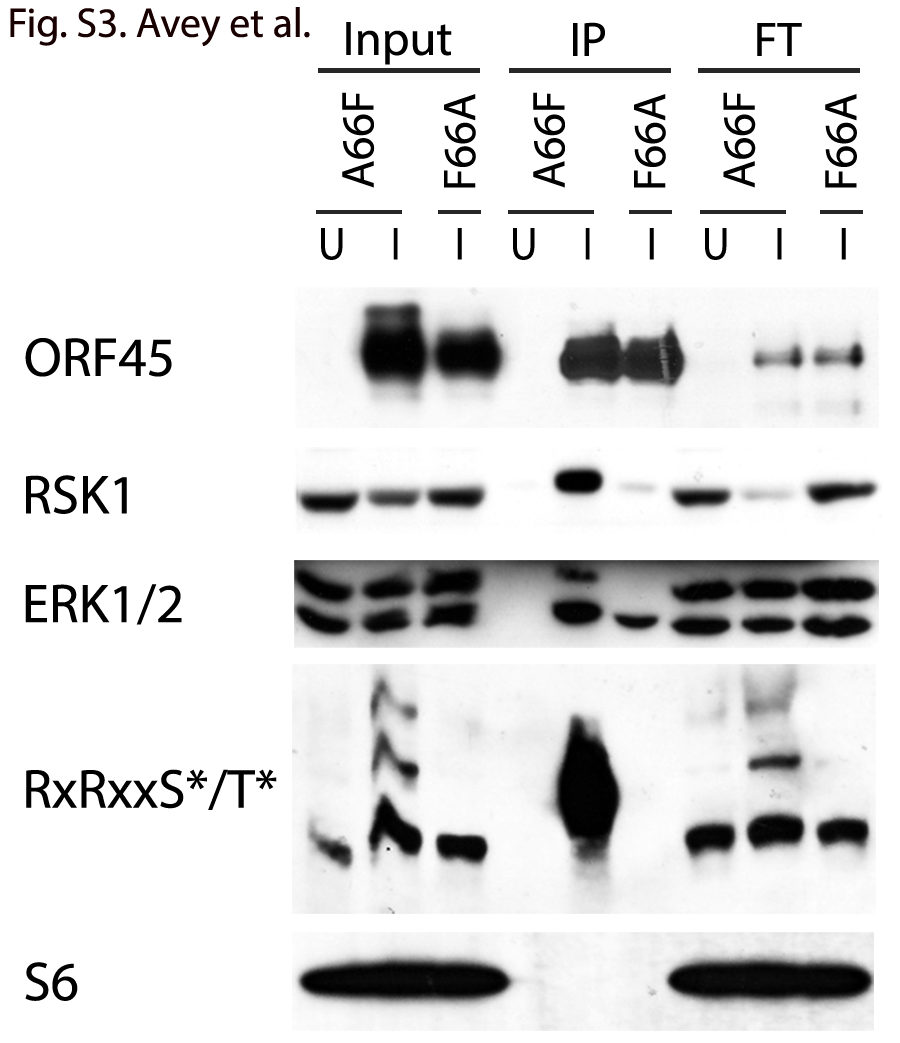

Supplement: S3 Fig — ORF45 was immunoprecipitated from iSLK.BAC16 A66F or iSLK.BAC16 F66A cells using 8B8 monoclonal antibody as described previously [15]. The inputs, eluates, and flow-throughs were analyzed by western blot with the indicated antibodies. (TIF) [file ppat.1004993.s005.tif]

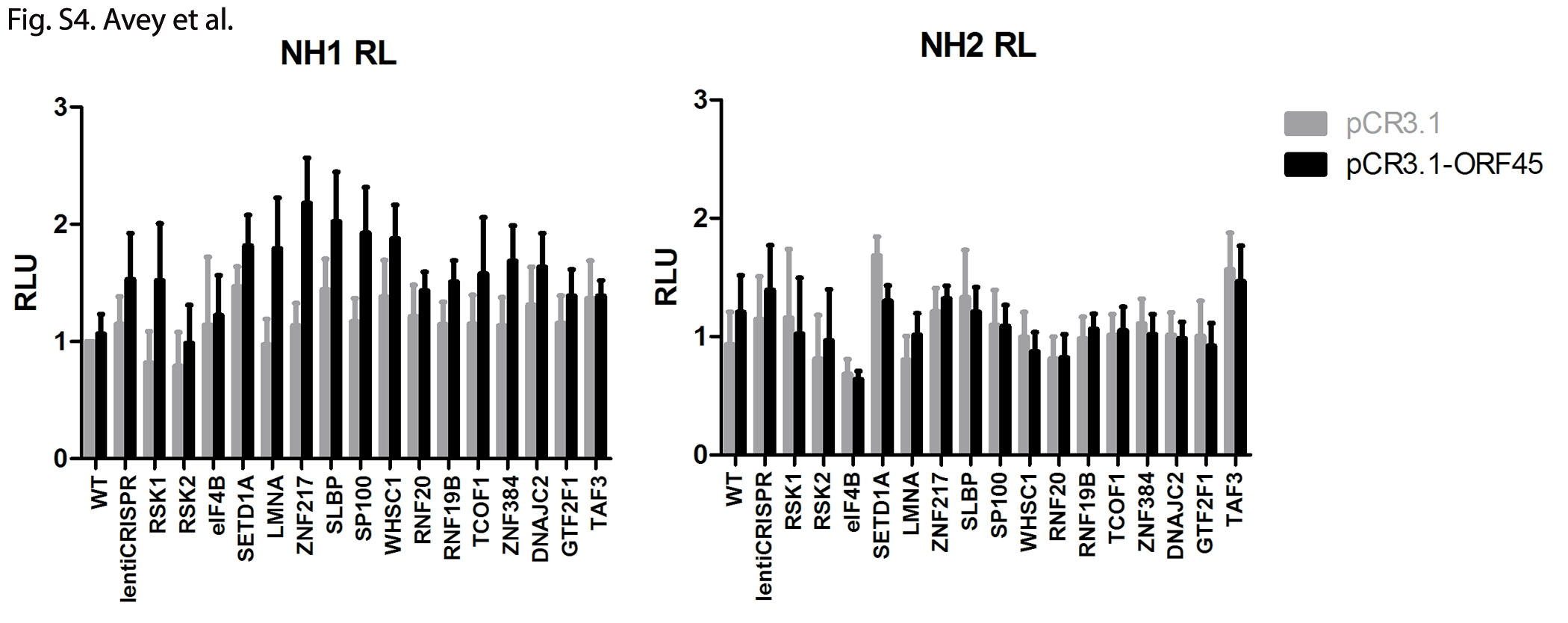

Supplement: S4 Fig — The same samples as in Fig 8A were analyzed for renilla luciferase activity. (TIF) [file ppat.1004993.s006.tif]
