# Supplementary material for: Phosphoproteomic Analysis of KSHV-Infected Cells Reveals Roles of ORF45-Activated RSK during Lytic Replication
Source: PLoS Pathog. 2015 Jul 2;11(7):e1004993. doi: 10.1371/journal.ppat.1004993 (PMC4489790; doi:10.1371/journal.ppat.1004993)
Supplement: S1 References — (DOCX) [file ppat.1004993.s008.docx]

**Supporting References:**

110. Roux PP, Shahbazian D, Vu H, Holz MK, Cohen MS, et al. (2007) RAS/ERK signaling promotes site-specific ribosomal protein S6 phosphorylation via RSK and stimulates cap-dependent translation. J Biol Chem 282: 14056-14064.

111. De Mesquita DD, Zhan Q, Crossley L, Badwey JA (2001) p90-RSK and Akt may promote rapid phosphorylation/inactivation of glycogen synthase kinase 3 in chemoattractant-stimulated neutrophils. FEBS Lett 502: 84-88.

112. Sutherland C, Leighton IA, Cohen P (1993) Inactivation of glycogen synthase kinase-3 beta by phosphorylation: new kinase connections in insulin and growth-factor signalling. Biochem J 296 ( Pt 1): 15-19.

113. Torres MA, Eldar-Finkelman H, Krebs EG, Moon RT (1999) Regulation of ribosomal S6 protein kinase-p90(rsk), glycogen synthase kinase 3, and beta-catenin in early Xenopus development. Mol Cell Biol 19: 1427-1437.

114. Bonni A, Brunet A, West AE, Datta SR, Takasu MA, et al. (1999) Cell survival promoted by the Ras-MAPK signaling pathway by transcription-dependent and -independent mechanisms. Science 286: 1358-1362.

115. Shimamura A, Ballif BA, Richards SA, Blenis J (2000) Rsk1 mediates a MEK-MAP kinase cell survival signal. Curr Biol 10: 127-135.

116. Anjum R, Roux PP, Ballif BA, Gygi SP, Blenis J (2005) The tumor suppressor DAP kinase is a target of RSK-mediated survival signaling. Curr Biol 15: 1762-1767.

117. Woo MS, Ohta Y, Rabinovitz I, Stossel TP, Blenis J (2004) Ribosomal S6 kinase (RSK) regulates phosphorylation of filamin A on an important regulatory site. Mol Cell Biol 24: 3025-3035.

118. Geraghty KM, Chen S, Harthill JE, Ibrahim AF, Toth R, et al. (2007) Regulation of multisite phosphorylation and 14-3-3 binding of AS160 in response to IGF-1, EGF, PMA and AICAR. Biochem J 407: 231-241.
